# Influence of Zn Content on the Corrosion and Mechanical Properties of Cast and Friction Stir-Welded Al-Si-Mg-Fe-Zn Alloys

**DOI:** 10.3390/ma18143306

**Published:** 2025-07-14

**Authors:** Xiaomi Chen, Kun Liu, Quan Liu, Jing Kong, Valentino A. M. Cristino, Kin-Ho Lo, Zhengchao Xie, Zhi Wang, Dongfu Song, Chi-Tat Kwok

**Affiliations:** 1 Department of Electromechanical Engineering, Faculty of Science and Technology, University of Macau, Macao, China; yc17474@um.edu.mo (X.C.); yc47948@um.edu.mo (K.L.); yc179237@um.edu.mo (Q.L.); yc279876@um.edu.mo (J.K.); vcristino@um.edu.mo (V.A.M.C.); fstkhl@um.edu.mo (K.-H.L.); 2 Zhuhai Institute of Advanced Technology, Chinese Academy of Sciences, Zhuhai 519085, China; 3 Institute of Applied Physics and Materials Engineering, University of Macau, Macao, China; 4 School of Mechanical and Automotive Engineering, South China University of Technology, Guangzhou 510641, China; zxie@scut.edu.cn (Z.X.); wangzhi@scut.edu.cn (Z.W.); songyuren1015@163.com (D.S.); 5 Guangdong Provincial Key Laboratory of Metal Toughening Technology and Application, Institute of New Materials Guangdong Academy of Sciences, Guangzhou 510650, China

**Keywords:** Al-Si-Mg-Fe-Zn alloys, friction stir welding, corrosion resistance, mechanical properties, microstructure

## Abstract

With the ongoing development of lightweight automobiles, research on new aluminum alloys and welding technology has gained significant attention. Friction stir welding (FSW) is a solid-state joining technique for welding aluminum alloys without melting. In this study, novel squeeze-cast Al-Si-Mg-Fe-Zn alloys with different Zn contents (0, 3.4, 6.5, and 8.3 wt%) were friction stir welded (FSWed) at a translational speed of 200 mm/min and a rotational speed of 800 rpm. These parameters were chosen based on the observations of visually sound welds, defect-free and fine-grained microstructures, homogeneous secondary phase distribution, and low roughness. Zn can affect the microstructure of Al-Si-Mg-Fe-Zn alloys, including the grain size and the content of secondary phases, leading to different mechanical and corrosion behavior. Adding different Zn contents with Mg forms the various amount of MgZn_2_, which has a significant strengthening effect on the alloys. Softening observed in the weld zones of the alloys with 0, 3.4, and 6.5 wt% Zn is primarily attributed to the reduction in Kernel Average Misorientation (KAM) and a decrease in the Si phase and MgZn_2_. Consequently, the mechanical strengths of the FSWed joints are lower as compared to the base material. Conversely, the FSWed alloy with 8.3 wt% Zn exhibited enhanced mechanical properties, with hardness of 116.3 HV_0.2_, yield strength (YS) of 184.4 MPa, ultimate tensile strength (UTS) of 226.9 MP, percent elongation (EL%) of 1.78%, and a strength coefficient exceeding 100%, indicating that the joint retains the strength of the as-cast one, due to refined grains and more uniformly dispersed secondary phases. The highest corrosion resistance of the FSWed alloy with 6.5%Zn is due to the smallest grain size and KAM, without MgZn_2_ and the highest percentage of {111} texture (24.8%).

## 1. Introduction

Lightweighting is a hot topic in the automobile industry. The body-in-white mass (BIW) referring to the automobile body is forty to fifty percent of the whole mass of the automobile. More lightweight BIW can reduce energy consumption and improve crash safety. Aluminum (Al) alloys replace the conventional steels as the BIW can reduce over forty percent of the mass [1]. Lightweight Al-Si casting alloys such as A356, A319 and A380 with low coefficient of thermal expansion, high electrical conductivity and corrosion resistance have been widely used in the automobile industry [2,3]. Nonetheless, the Al-Si casting alloys are rarely used on BIW. The main reason is that the materials are difficult to manufacture complex, thin and large structural components, and are complex and expensive [3,4]. In the automotive industry, there are many non-standard Al alloys with different specific properties. In this study, friction stir welding (FSW) of novel squeeze-cast Al-Si-Mg-Fe-Zn alloys with different Zn contents (0, 3.4, 6.5, and 8.3 wt%) were investigated. Squeeze casting combines casting and forging in manufacturing process with high efficiency and accurate forming [5]. Addition of various alloying elements in the Al alloys is for different purposes. Proper Fe content added to the alloys is to avoid the mold sticking [2]. However, Fe can weaken the mechanical properties due to the formation of hard and brittle β-AlFeSi phases and low bonding strength with the α-Al matrix [6,7,8]. To reduce the influence of Fe, Mn is often added to the materials to form the Chinese script α-AlFeMnSi phases which avoid sticking with the mold during casting and also improve the corrosion resistance and mechanical strength [7,8,9]. The appropriate addition of Sr can refine the Si and change the shape from sheet to spherical shape to improve the mechanical properties [10,11]. In addition, suitable Ti in the alloys can help to refine α-Al matrix [12,13,14]. Moreover, the proper addition of Mg can form the precipitations (MgZn_2_ and Mg_2_Si) to improve the mechanical properties [15,16]. The suitable Zn content also can generate the strengthening precipitates MgZn_2_ and induce Mg and Si to form more Mg_2_Si [17,18,19,20]. Cao et al. [21] found that Zn content with less than 1% can slightly refine the microstructure of as-cast AlMgSiCu alloy, and more segregation at the grain boundaries of α-Al, and the precipitated phases increase with the increase in Zn content in the alloy. It was also observed that the addition of Zn did not affect the phase compositions and morphology of the as-cast alloy and no Zn-rich phases formed [22], which may be related to the high solid solubility of Zn in Al (at 381 °C), Al can dissolve 83.1 wt% of Zn. Al can only dissolve about 2 wt% of Zn at room temperature. Tang [23] pointed out that with the addition of Zn from 20 wt% to 40 wt% in 7XXX Al alloy, grain size can be refined from 45 to 20 μm, and non-equilibrium η-MgZn_2_ at the grain boundaries can be increased from 9% to 28%, leading to increase in hardness from 75 to 140 HV, and improvement of yield strength from 320 MPa to 430 MPa but decline in elongation from 7% to 3%. In addition, the increase in Zn content can lower the melting temperature and improve the fluidity of the alloy [23,24]. Zhang et al. [25] reported that Zn could dissolved and uniformly distributed in the α-Al matrix. It is also mentioned that Zn can dissolve in α-Al and Mg_2_Al_3_ in Al-Zn-Mg alloy at the same time, and the formation of strengthened phases MgZn_2_ and τ phase (Al_2_Zn_3_Mg_3_) reduces the diffusion ability of Mg atoms, inhibits Mg diffusion and discontinuous distribution of Mg_2_Al_3_, thus significantly improving the tensile strength and stress-corrosion resistance. Also, the Si phase can be refined from coarse irregular shape to the round granular shape with the addition of Zn, which significantly increases the hardness but reduces the machinability of the alloy. Adding a certain amount of Zn to 3003 (Al-Mn-Fe-Si alloy) can enhance the corrosion resistance between the fin and the diversion pipe [26]. While Zn in 3003 alloy can induce the transformation of Al_6_(Fe, Mn) to α-Al_12_(Fe, Mn)_3_Si which is finer and more uniform distributed after heat treatment, leading to enhanced strength [27]. The hardness of cast Al-6061 with 4 wt% Zn is the highest (157 HV) [28], because of the MgZn_2_ precipitate and fine recrystallized grains. The ultimate tensile stress of the cast Al-6061 (198.1 MPa) increases with the increase in Zn content from 214.9 MPa (1 wt%) to 265.0 MPa (4 wt%) and then decreases with the further increase in Zn content from 4 wt% to 5 wt% (244.9 MPa). The addition of a small amount of Mg (0.1~1 wt%) and Zn (0.1~0.55 wt%) to the Al 1XXX alloys led to the formation of β-(Al_3_Mg_2_), τ-(Mg_32_(Al, Zn)_49_) phases [29]. The formation of phases at the grain boundaries of the Al-Mg-Zn alloy resulted in the transformation from pitting corrosion to intergranular corrosion, thereby enhancing the overall corrosion resistance. The addition of suitable amount of Zn could enhance the corrosion resistance of the Al-6Si-3Cu-xZn-T6 alloy, but excessive Zn content would reduce the corrosion resistance. As the excess Zn content increased the corrosion rate by adsorbing Zn ions into the passivation film and making it porous [30]. The Al-6Si-3Cu alloy with 0.97 wt% Zn has the highest corrosion resistance, which is better than that of the alloy without Zn [31]. The addition of Zn could form a Zn-rich area around the Al and weakened the corrosion resistance between the Al_2_Cu and the Al matrix. However, excess Zn content altered the microstructure of the Al_2_Cu phase which became the electron transfer channel between the Si phases, resulting in decreased corrosion resistance.

**Figure 1:**
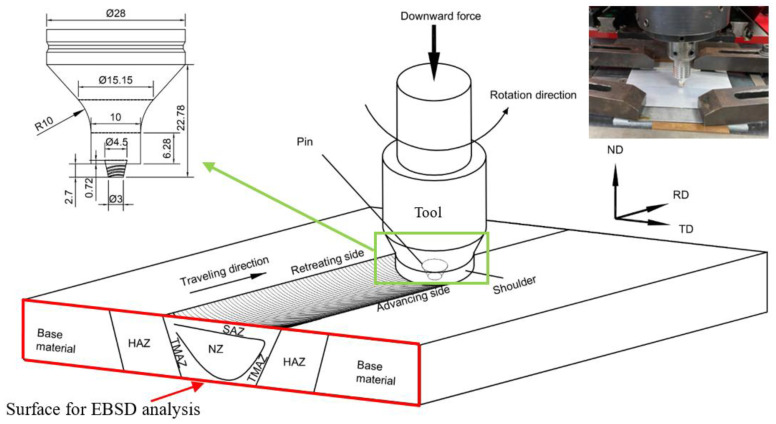
Setup for FSW.

Weld defects, including cracks, slag inclusions and pores, are often produced via tungsten inert gas (TIG) and melt inert-gas welding (MIG), leading to poor properties of the joints [32,33]. While FSW is an efficient and commonly used solid-state joining method for joining Al alloys used in aerospace, ships, automobiles, and rail transit. Preheat and melting of Al alloys are not necessary in FSW [33,34]. FSWed joints possess high quality and strength [35,36]. During FSW, the materials undergo severe plastic deformation (SPD) [37] and experience dynamic recrystallization (DRX), leading to refinement of microstructure, lower residual stress, improved dimensional stability, and enhanced mechanical properties [32,33,38,39,40]. Figure 1 shows the typical FSWed joint with five different areas formed at different temperature [41,42] and flow [43,44], including the weld nugget zone (NZ), shoulder-affected zone (SAZ), thermo-mechanically affected zone (TMAZ) and the heat-affected zone (HAZ), and also unaffected base materials (BM).

The heat input is very important for the FSW process, since the materials in NZ undergo severe agitation and experience thermal cycle effect under stirring and friction of the tool [45]. The peak temperature T_peak_ (°C) indicates the heat generation and input during FSW. It is about 70% of the melting temperature of the alloys [40,46]. FSW technology is essentially a welding method that uses frictional heat as the welding heat source, so the use of heat transport (q_E_) to evaluate the quality of the joint is the most direct and effective [47]. The equation of heat transport (q_E_) is followed by:

q_E_ = kω/ʋ (1)

where k is the heat input coefficient, which is dependent on the shape of tool and related to friction coefficient and welding pressure; ω is the rotation speed; and ʋ is the traveling speed. In the present study, the same FSW parameters (ω = 800 rpm, ʋ = 200 mm/min) were used, so only k influenced the q_E_.

There is limited understanding of how Zn affects the microstructure, mechanical properties, and corrosion resistance of FSWed Al-Si-Mg-Fe-Zn alloys. Existing literature often reports conflicting results regarding the optimal Zn content for achieving the best weld quality and performance, indicating a need for further investigation. Understanding the role of Zn can lead to enhanced mechanical properties in welded joints, such as increased strength, ductility, and hardness. Insights gained from the present work can inform the design of Al-Si-Mg-Fe-Zn alloys for specific applications, particularly in industries requiring lightweight and high-strength materials. Moreover, FSW of Al-Si-Mg-Fe-Zn alloys can reduce porosity and improve the overall quality of the welds.

Heat, as well as force, affects the material flow and hence the microstructure of the FSWed joint. During FSW, the strengthening precipitate (MgZn_2_) in the alloys may become coarser or dissolve in the Al matrix, which may form the softening area and reduce the strength [48,49]. On the other hand, FSW of AA 7075 showed significant refinement of the α-Al grains and MgZn_2_ in the NZ [48]. Many nano-sized MgZn_2_ precipitates were uniformly distributed in the α-Al of AA7449, but these precipitates were completely dissolved after FSW [50]. Kesharwani et al. [51] investigated the effect of addition of Zn powder on the surface of AA 6061 during FSW. Under high temperature generated by the stirring tool during FSW, the solubility of Zn in the α-Al increased. The dissolved Zn atoms in the α-Al acted as solute atoms and caused the strain field. The strain field hindered the movement of dislocations. Therefore, dislocations encountered greater resistance in passing through the strain field, and the strength of the joint was enhanced [51,52].

Adding suitable amount of Zn is beneficial for mechanical properties [24,53,54,55] and corrosion resistance [29,30,31,56] of the Al-Si alloys. In the literature, there is no study on the effect of Zn contents on the FSWed Al-Si-Mg-Fe-Zn alloys. In the present study, FSW of squeeze-cast Al-Si-Mg-Fe-Zn alloys with different Zn contents (0, 3.4, 6.5 and 8.3 wt%) was investigated and the corrosion and mechanical properties, and microstructure of the FSWed joints were evaluated.

## 2. Materials and Methods

### 2.1. Materials

The chemical compositions of Al-Si-Mg-Fe-0%Zn, Al-Si-Mg-Fe-3.4%Zn, Al-Si-Mg-Fe-6.5%Zn, and Al-Si-Mg-Fe-8.3%Zn in as-cast condition are shown in Table 1. The Zn contents of the specimens increased successively as follows: 0 wt%, 3.4 wt%, 6.5 wt% and 8.3 wt% while Mg content was kept constant (i.e., 0.5 wt%).

### 2.2. Different Scanning Calorimetry (DSC)

The melting temperature and enthalpy of the as-cast Al-Si-Mg-Fe-Zn alloys and the NZ of the FSWed joints were tested using differential scanning calorimetry (DSC, Themys One, KEP Technologies, Caluire, France). The test coupons were cut from the specimens with about 10 mg and kept into a 85-μL alumina crucible, which was put in the furnace with Argon flowing at a rate of 20 mL/min. In the beginning, the specimens were raised from room temperature to 30 °C at a heating rate of 5 K/min, then heated from 30 to 750 °C at 10 K/min, and finally cooled from 750 °C to 30 °C at cooling rate of 30 K/min.

### 2.3. Friction Stir Welding

Figure 1 reveals the schematic diagram of setup of the FSW machine (China FSW Center, FSW-TS-M16, Bejing, China). Two similar plates, each with dimensions of 180 × 90 × 3 mm^3^, were joined with the FSW machine at traveling speed of 200 mm/min and rotation speed of 800 rpm. FSW of AA6063 aluminum alloy was carried out by Chinchanikar and co-workers [57] for investigating the effects of rotational speeds (ω, 385–960 rpm) and translational speeds (*v*, 18–45 mm/min) on tool forces, micro-hardness and surface roughness of the weld bead. Low tool forces at high rotational and translational speeds were due to the increase in temperature at the weld bead resulting in softening of the material. In addition, high surface roughness obtained at low rotational speed was owing to less heat generation at weld bead and fast solidification rate of the softened material. At high rotational speed, large amount of heat generation caused excessive release of stirred material to the upper surface of the weld bead resulting in low micro-hardness. However, the micro-hardness was found to be less significantly affected by FSW parameters. On the other hand, FSW of AA2024-T6 aluminum alloy was performed by Farhang et al. [58] using different and translational speeds (25–31.5 mm/min) and rotational speeds (1120–1600 rpm) for improving the mechanical strength and microstructure properties. With the increase in rotational speed, the grain size of the weld bead increased, and more homogeneous distribution of secondary phase was obtained. Moreover, by increasing both translational and rotational speeds, the hardness of the TMAZ and NZ could increase to the hardness of the base metal. In the present study, preliminary trials of the different FSW parameters, i.e., translational and rotational speeds, were attempted. According to the work of Cahalan et al. [59], the heat input is proportional to the ratio of rotational speed to translational speed (ω/*v*). Compared with the ratios reported by Chinchanikar et al. [57] (ω/*v* = 8.5 to 53.3) and Farhang et al. [58] (ω/*v* = 35.6 to 64), the optimized parameters chosen in the present work were a translational speed of 200 mm/min and a rotational speed of 800 rpm (ω/*v* = 4), based on the observation of a visually sound weld, microscopically defect-free weld bead, fine grained structure, homogeneous secondary phase distribution, low roughness. In addition, the relatively high translational speed was used (hence lower ω/*v*) as the processing efficiency is also a major concern. The tool for FSW was made of H13 tool steel with a length of 2.7 mm. The pressing depth of the shoulder to the material was 0.1 mm, so the plunged depth of tool was 2.8 mm with plunged speed of 30 mm/min and plunged delay time of 2 s. As the FSW process completed, the tool was stayed for 1 s delayed before lifting up with speed of 50 mm/min and height of 20 mm. During FSW, the tool was tilted at 2.5°.

### 2.4. Analysis of Microstructure

The as-cast alloys and the top surface of the FSWed joints were prepared to the dimensions of 15 × 10 × 3 mm^3^ and ground with the emery paper of #2500. Then they were observed using the X-Ray diffractometer (XRD, MiniFlex 600, Rigaku, Tokyo, Japan, Cu-K_α_ radiation) operated at 40 kV and 15 mA. The diffraction angle (2θ) was scanned from 20° to 140° at a rate of 0.5°/min for phase identification. Furthermore, they were also analyzed by X-ray photoelectron spectroscopy (XPS, Thermo Scientific K-Alpha, East Grinstead, UK) operated with Al-K_α_ radiation (hv-1486.6 eV) at beam spot of 400 μm. The full spectrum bandwidth was 100 eV with step size of 1 eV and the narrow spectrum bandwidth was 60 eV with step size of 0.1 eV. The absolute binding energy was normalized by C (1s) spectral line with binding energy of 284.8 eV.

The cross-section of the FSWed joints were cut to the dimensions of 30 × 7 × 3 mm^3^ by wire electrical discharge machining (WEDM). Then the specimens were ground and further polished with the 1-μm diamond paste. Furthermore, the polished specimens were etched for 20 s with 35% NaOH solution. The etched specimens were investigated using optical microscope (Leica, DMI3000 M, Durham, NC, USA).

The specimens were studied using field emission scanning electron microscope (FESEM, Zigma, Zeiss, Oberkochen, Germany) with the EDS (X-Max, Oxford Instrument, Oxford, UK) and EBSD (NordlysNano, Oxford Instrument, Oxford, UK) detectors. The cross-sections of the FSWed joints were polished with the vibratory polishing machine (VibroMet 2, Buehler, Lake Bluff, IL, USA) with the 0.05-μm colloidal silica slurry at frequency of 40–60 Hz for 2 to 3 h. The -RD direction in EBSD was the normal direction as shown in Figure 1. The EBSD data was analyzed using the software ‘Azteccrystal 3.1’ (Oxford Instrument, UK). Using EBSD, the distribution of kernel average misorientation (KAM) was measured. The average KAM (KAM_ave_) is directly proportional to the density of geometrically necessary dislocations (*ρ*_GND_) [60,61]. The *ρ*_GND_ has the potential to alter the slip system in Al-alloys with high stacking fault energy [62] and serves as a measure of residual stress or internal energy [63].

Furthermore, as-cast Al-Si-Mg-Fe-8.3%Zn and the NZ of the FSWed Al-Si-Mg-Fe-8.3%Zn were investigated using the field emission transmission electron microscope (TEM) (Talos F200X S/TEM, Thermo Fisher Scientific, Waltham, MA, USA) at 200 kV investigated by a Ceta CMOS camera. The ion beam milling instrument (Gatan PIPS II 695, Gatan, Inc., Pleasanton, CA, USA) was used to prepare the TEM thin foils at 5, 4 and 3 keV successively. In addition, the ImageJ 1.53a software was used to measure the grain size of the precipitates.

### 2.5. Hardness and Tensile Tests

**Figure 2:**
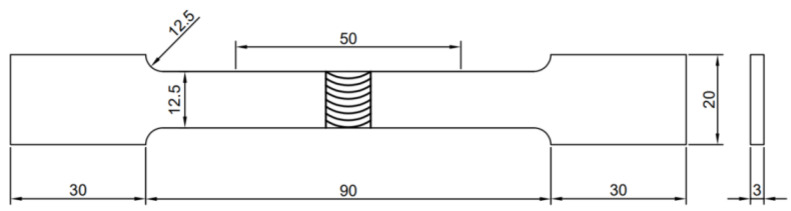
Dimensions of the specimens for tensile tests (in mm).

The Vickers hardness of FSWed joints were investigated using Vickers hardness tester (Qness 60A EVO, QATM, Mammelzen, Germany) with 200 g load and 10 s across the cross-sections (about 1 mm away from the top surface). The tensile tests of as-cast Al-Si-Mg-Fe-Zn alloys and their FSWed joints were conducted using the universal tensile testing machine (E45, MTS, Danvers, MA, USA), and the specimens were cut into the dimensions according to the ASTM standard E8M-04 [64] as shown in Figure 2. After the tensile test, the fracture morphology was observed using the scanning electron microscope (S-3400N, Hitachi, Tokyo, Japan).

### 2.6. Electrochemical Measurements

The polarization tests of the as-cast alloys and the NZ of the FSWed joints were measured using three-electrode electrochemical workstation (Princeton Versa STAT 3, Oak Ridge, TN, USA). The saturated calomel electrode (SCE, 0.242 V versus SHE) was the reference electrode (RE) at 25 °C, and a platinum plate was the counter electrode (CE). The specimens as the working electrode (WE) with size of 15 × 15 × 3 mm^3^ were mounted in the cold-curing epoxy resin. The test solution was 3.5 wt% NaCl and was placed in an electronic water bath at 25 ± 1 °C. After measuring the open-circuit potential (OCP) test for 30 min, the electrochemical impedance spectroscopy (EIS) and polarization tests were carried out separately. The EIS tests were measured at OCP perturbed at ±10 mV and the frequency from 100 kHz to 0.01 Hz. The software ‘Zview 3.1’ was used to analyze the EIS results. In addition, polarization tests were carried out from −0.6 to +0.6 V_SCE_ (vs OCP) with a rate of 1 mV/s. For each specimen, the tests were repeated three times. After the electrochemical tests, the corroded surface was observed using SEM and EDS.

## 3. Results and Discussion

### 3.1. DSC

**Figure 3:**
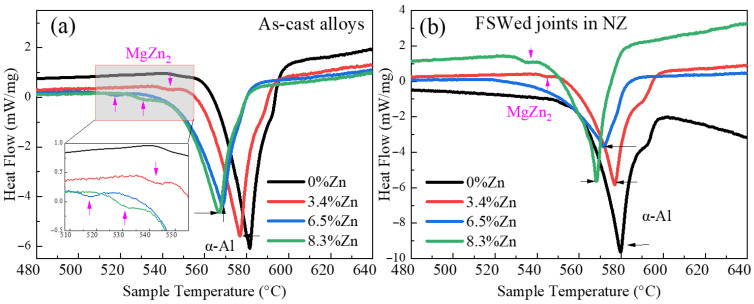
DSC curves of (**a**) as-cast specimens and (**b**) NZ of FSWed joint.

Figure 3 reveals the DSC curves and Table 2 indicates the analyzed DSC results of the as-cast alloys and the NZs of the FSWed joints. Two absorption peaks can be observed, one is the melting temperature of α-Al, and the other one is the low solidus temperature of the eutectic phase (i.e., MgZn_2_) [65]. Al-Si-Mg-Fe-0%Zn without Zn (MgZn_2_) has the highest melting temperature before and after FSW. Therefore, a higher heat input is required in Al-Si-Mg-Fe-0%Zn during FSW. The following section of DSC mainly discusses the samples containing Zn. While the melting temperature of the as-cast alloys and NZs of the FSWed joints decreases as the Zn content increases. According to thermodynamics, the driving force for MgZn_2_ dissolution is the change in Gibbs free energy:

△G = △H − *T*△S (2)

where △H is the change in enthalpy, *T* is the temperature and △S is the change in entropy [66]. When dissolution reaches equilibrium at melting point, △G = 0 and △H = *T_m_* △S. △S is directly proportional to ln$C_{\;MgZn2}$ in ideal solid solution, so the solute concentration of MgZn_2_ can be calculated by the equation:

(3) $$C_{\;MgZn2}\approx C_{0}exp(-\frac{△H}{RT})$$

where *C*_0_ is the pre-exponential factor, *R* is the gas constant and *T_m_* is the melting temperature [67]. The greater the enthalpy of melting (DH), because a higher *T_m_* indicates stronger bonds, and breaking these stronger bonds requires more energy.

The solidus temperature of MgZn_2_ in as-cast Al-Si-Mg-Fe-3.4%Zn is the highest (542 °C), followed by as-cast Al-Si-Mg-Fe-8.3%Zn (530.8 °C), and the as-cast Al-Si-Mg-Fe-6.5%Zn (517.3 °C) is the lowest. The content of eutectic Al-Si in the as-cast Al-Si-Mg-Fe-6.5%Zn are the highest. During the casting process, the liquid phase participated in the polyeutectic reaction, i.e.,

L → eutectic (α-Al + Si) + MgZn_2_

rather than precipitating the primary α-Al phase (high melting point). In addition, 6.5% Zn is close to the solubility limit of the alloy, and a more solid solution of Zn to the α-Al matrix can induce more lattice distortion and then the Al-Al chemical bond is weakened, leading to lower melting temperature. However, addition of 8.3%Zn to the alloy leads to more MgZn_2_ and other intermetallic compounds such as β-(Mg_2_Al_3_) [68] with higher melting temperature.

In addition, the melting enthalpy of the endothermic peak reveals the content of phases, so the eutectic phase (MgZn_2_) in the as-cast alloys increases with the increase in Zn content. After FSW, MgZn_2_ is not detected in FSWed Al-Si-Mg-Fe-6.5%Zn because of the lowest melting point of MgZn_2_ in the as-cast Al-Si-Mg-Fe-6.5%Zn and then the MgZn_2_ easily dissolved as the solid solution after FSW. The contents of MgZn_2_ in the NZ of the FSWed Al-Si-Mg-Fe-3.4%Zn and FSPed Al-Si-Mg-Fe-8.3%Zn are slightly higher than that in the as-cast ones, possibly caused by limited redissolution and generation of new precipitation during FSW.

The calculation of the peak temperature (T_peak_) during FSW is 397~403 °C lower than the melting temperature of MgZn_2_, indicating no secondary phases were melted. At T_peak_, Mg_2_Si and MgZn_2_ may be dissolved into α-Al matrix due to higher solubility at high temperature, according to the phase diagrams of Al–Mg_2_Si [69] and Al–MgZn_2_ [70]. But it is possible that they will re-precipitate during cooling. Thus, the solutionizing effect on the NZ of FSWed Al-Si-Mg-Fe-6.5%Zn is more significant than the precipitation effect, while solutionizing effect in the FSWed Al-Si-Mg-Fe-3.4%Zn or FSWed Al-Si-Mg-Fe-8.3%Zn is less profound.

### 3.2. Microstructure Analysis of FSWed Joints

**Figure 4:**
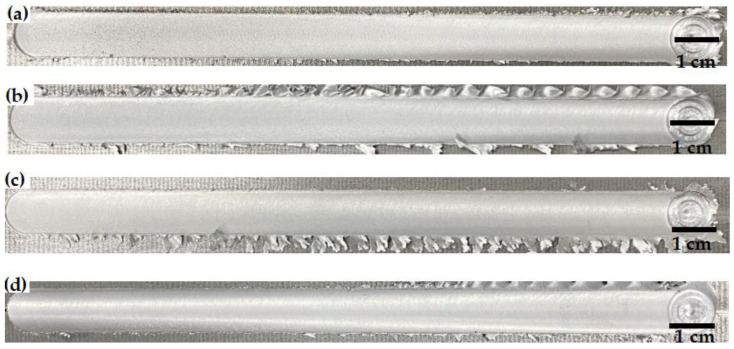
FSWed joints of (**a**) Al-Si-Mg-Fe-0%Zn, (**b**) Al-Si-Mg-Fe-3.4%Zn, (**c**) Al-Si-Mg-Fe-3.4%Zn, and (**d**) Al-Si-Mg-Fe-8.3%Zn.

Figure 4 shows the top views of the FSWed joints of Al-Si-Mg-Fe-0%Zn, Al-Si-Mg-Fe-3.4%Zn, Al-Si-Mg-Fe-6.5%Zn and Al-Si-Mg-Fe-8.3%Zn. The top surface of the FSWed joints has a scaly shape and no cracks, indicating the material in plastic state with perfect fluidity and formability under the rotating of the tool during FSW. There are slight weld flash on the surface at the edge of the weld track owing to the slight difference in thickness of the plates [47].

**Figure 5:**
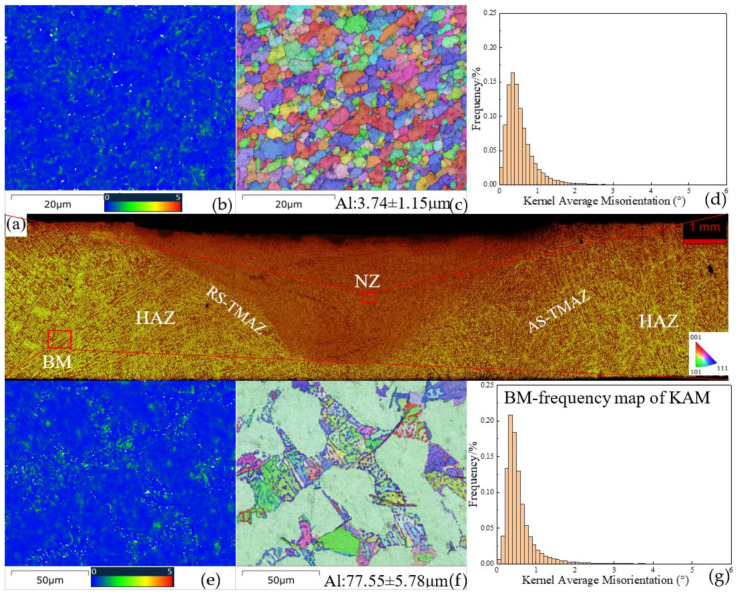
Cross-sectional microstructures of FSWed Al-Si-Mg-Fe-0%Zn; (**a**) overall view; (**b**) NZ-KAM, (**c**) NZ-BC + IPF||Z + GB, (**d**) NZ-frequency map of KAM, (**e**) BM-KAM, (**f**) BM-BC + IPF||Z + GB, (**g**) BM-frequency map of KAM.

Figure 5 shows the microstructure of the cross-section of the NZ and BM of the FSWed Al-Si-Mg-Fe-0%Zn with the KAM and BC + IPF||Z + GB. The texture of the as-cast Al-Si-Mg-Fe-0%Zn is mainly <101>||Z_0_. The grain size of α-Al in the as-cast Al-Si-Mg-Fe-0%Zn is about 77.55 ± 5.78 μm and the KAM in NZ and BM are small. The lowest KAM in the NZ and BM of FSWed Al-Si-Mg-Fe-0%Zn is due to absence of Zn. As Zn can dissolve in the Al matrix causing lattice distortion, which can also increase the KAM. After FSW, the grain size is refined and the LAGBs decrease in the NZ of Al-Si-Mg-Fe-0%Zn due to the SPD and DRX.

**Figure 6:**
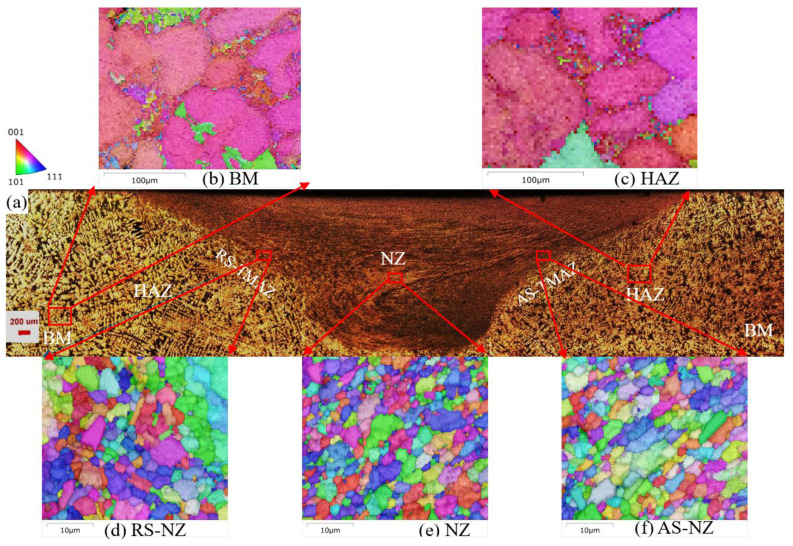
Cross-sectional microstructures of FSWed Al-Si-Mg-Fe-3.4%Zn; (**a**) overall view; (**b**) BM, (**c**) HAZ, (**d**) RS-NZ, (**e**) NZ, (**f**) AS-NZ.

**Figure 7:**
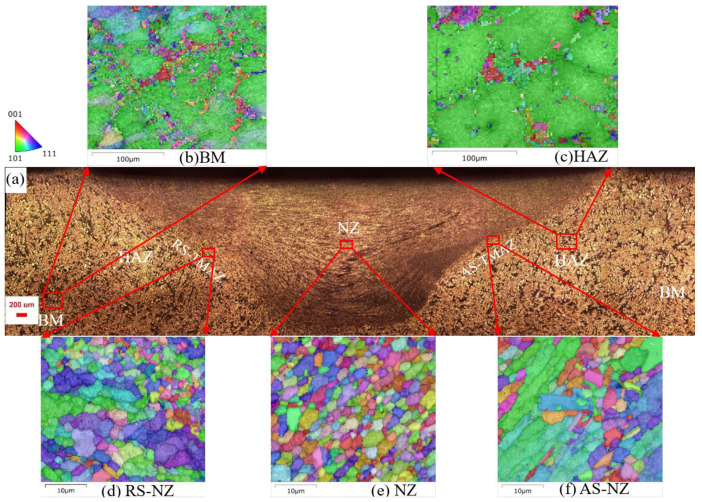
Cross-sectional microstructures of FSWed Al-Si-Mg-Fe-6.5%Zn; (**a**) overall view; (**b**) BM, (**c**) HAZ, (**d**) RS-NZ, (**e**) NZ, (**f**) AS-NZ.

**Figure 8:**
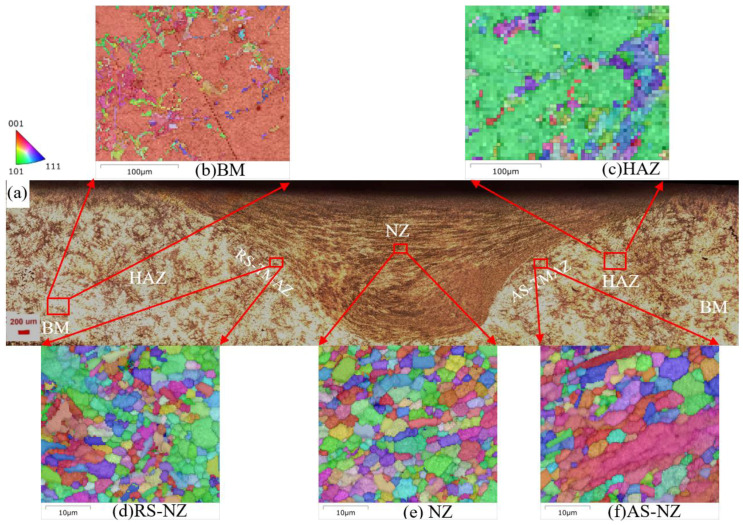
Cross-sectional microstructures of FSWed Al-Si-Mg-Fe-8.3%Zn; (**a**) overall view; (**b**) BM, (**c**) HAZ, (**d**) RS-NZ, (**e**) NZ, (**f**) AS-NZ.

**Figure 9:**
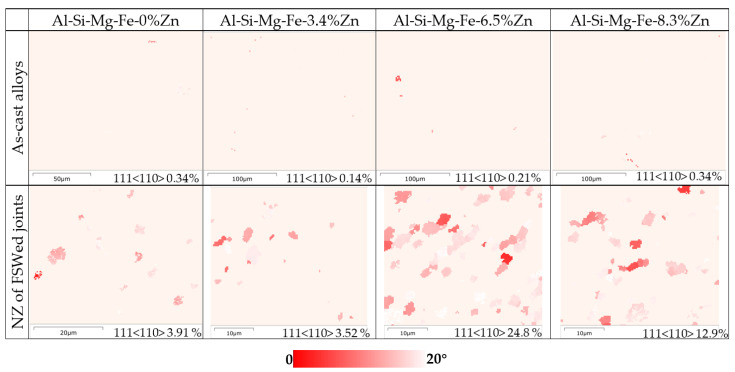
Distribution map of α-Al {111}<110> texture of different specimens.

Figure 6, Figure 7 and Figure 8 show the microstructure of the cross-section of the FSWed joints with different regions, including the retreating side (RS), NZ (center), AS-NZ, advancing side (AS), TMAZ, HAZ, and BM. Figure 9 shows the distribution map of α-Al {111} <110> texture of different specimens. From Figure 6a, Figure 7a and Figure 8a, the textures of the as-cast Al-Si-Mg-Fe-3.4%Zn and Al-Si-Mg-Fe-8.3%Zn are mainly <114>||Z_0_, while the texture of the as-cast Al-Si-Mg-Fe-6.5%Zn is also mainly <101>||Z_0_, since the squeeze-cast alloys are in form of sheets with the squeeze force acting in one direction. The difference in the texture among the as-cast alloys caused by the different lattice distortion induced by Zn. It was reported that the stainless steels experience SPD and DRX during FSW process, resulting in {111}<110> ideal simple shear texture [71,72,73,74,75,76] which is parallel to the slip system of fcc α-Al [77]. From the EBSD results, the texture of the slip system {111}<110> increases by about 3.4% in FSWed Al-Si-Mg-Fe-3.4%Zn and FSWed Al-Si-Mg-Fe-0%Zn, 24.6% in FSWed Al-Si-Mg-Fe-6.5%Zn and 12.56% in FSWed Al-Si-Mg-Fe-8.3%Zn, as shown in Figure 9. FSWed Al-Si-Mg-Fe-6.5%Zn has highest {111}<110> texture, indicating more SPD and DRX during FSW, due to the decrease in MgZn_2_ content. From the DSC results, MgZn_2_ was not detected in FSWed Al-Si-Mg-Fe-6.5%Zn, but the content of MgZn_2_ in Al-Si-Mg-Fe-3.4%Zn and Al-Si-Mg-Fe-8.3%Zn increased after FSW. The decrease in MgZn_2_ content increased the material flow and the dissolution of Mg and Zn into the α-Al matrix caused lattice distortion, resulting in the material in Al-Si-Mg-Fe-6.5%Zn moving more towards the shear texture orientation during FSW.

**Figure 10:**
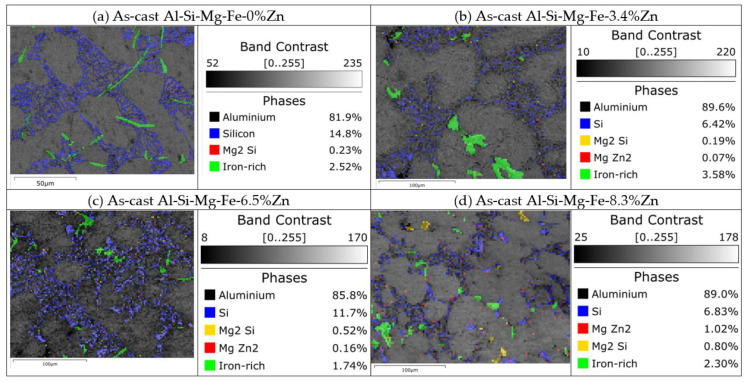
Phases and band contrast maps of (**a**) as-cast Al-Si-Mg-Fe-0%Zn, (**b**) as-cast Al-Si-Mg-Fe-3.4%Zn, (**c**) as-cast Al-Si-Mg-Fe-6.5%Zn, and (**d**) as-cast Al-Si-Mg-Fe-8.3%Zn.

**Figure A1:**
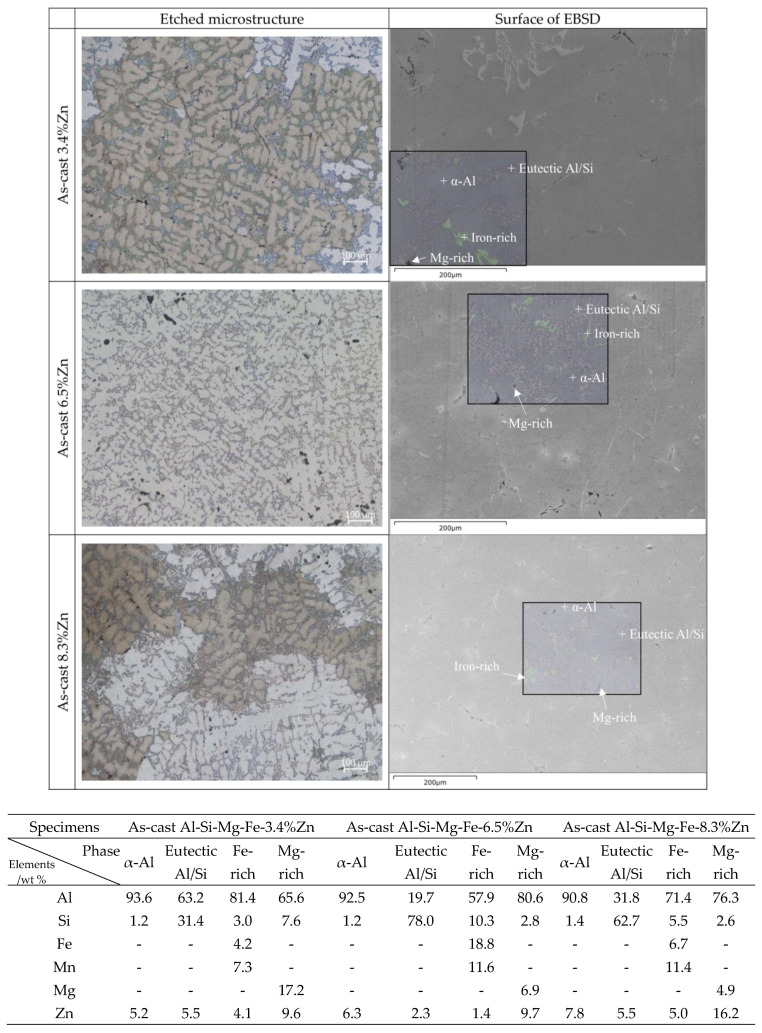

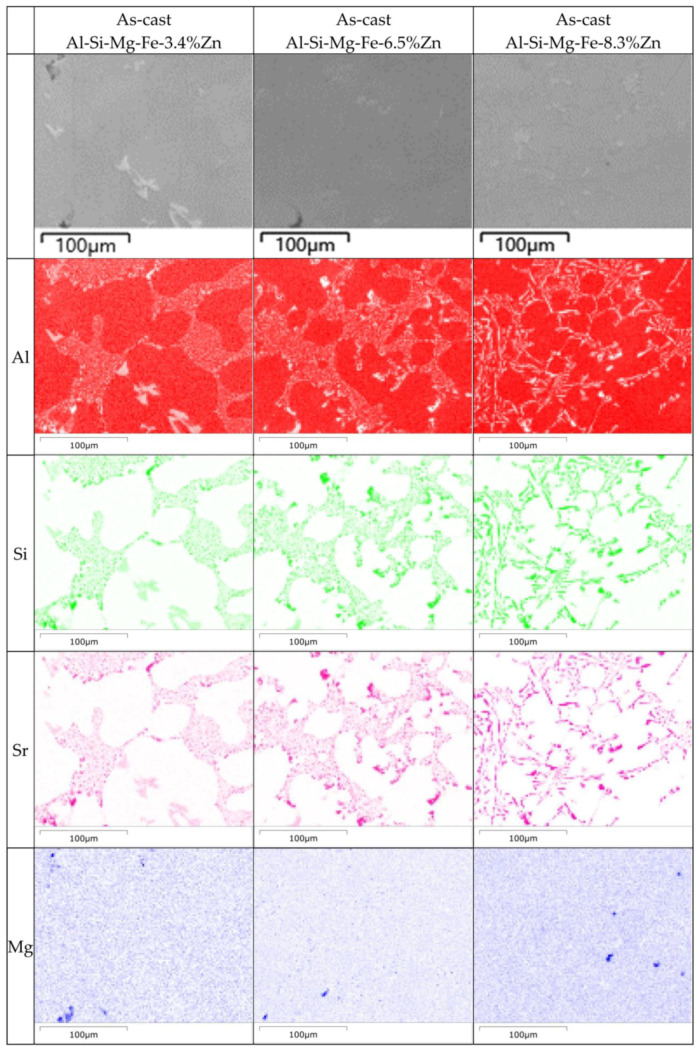

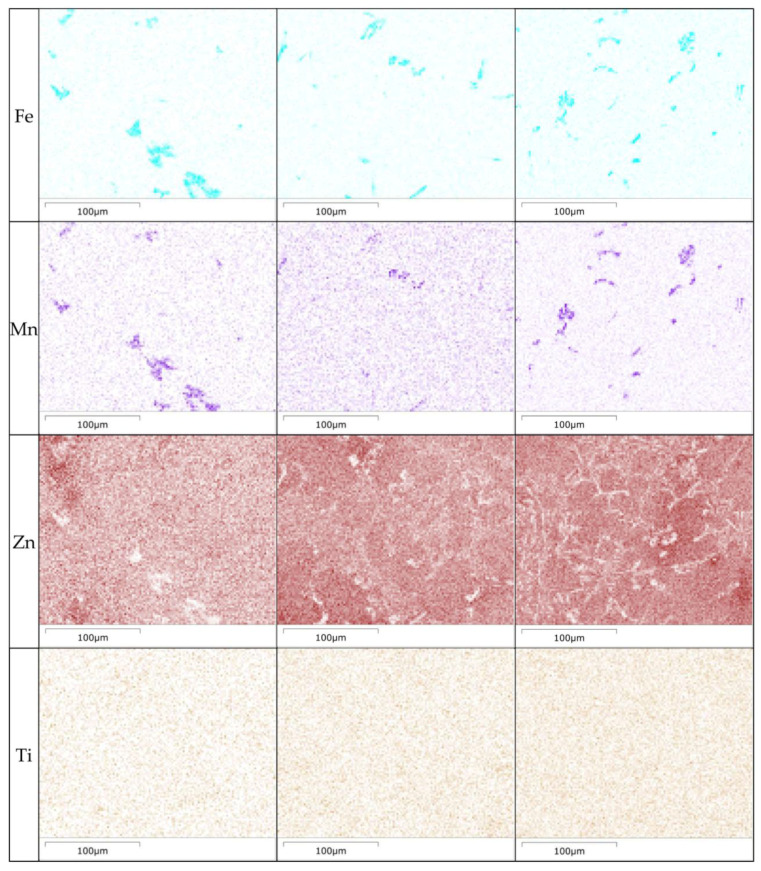
SEM images and EDS maps of as-cast alloys.

Figure 10 shows the phase and band contrast maps of the BM (the as-cast alloys). α-Al dendrites, eutectic Al-Si, Fe-rich phases, Mg_2_Si and MgZn_2_ can be observed. The shape of the Fe-rich phases in the as-cast Al-Si-Mg-Fe-3.4%Zn shows the large dendritic network. With the increase in Zn content, Fe-rich phases are finer and evenly distributed [27]. Zn in α-Al can promote the precipitation of Mn, which is more conducive to promote the transformation of the Al_6_(Fe,Mn) into the α-Al_12_(Fe,Mn)_3_Si (the Fe-rich phase) [27]. From the table in Figure A1, the Fe-rich phase is found to be α-Al_12_(Fe,Mn)_3_Si. Meanwhile, Zn can reduce the vacancy concentration in the matrix, restrict the movement of vacancies, slow down the growth of the precipitates, and make the α-Al_12_(Fe, Mn)_3_Si more dispersed.

**Figure 11:**
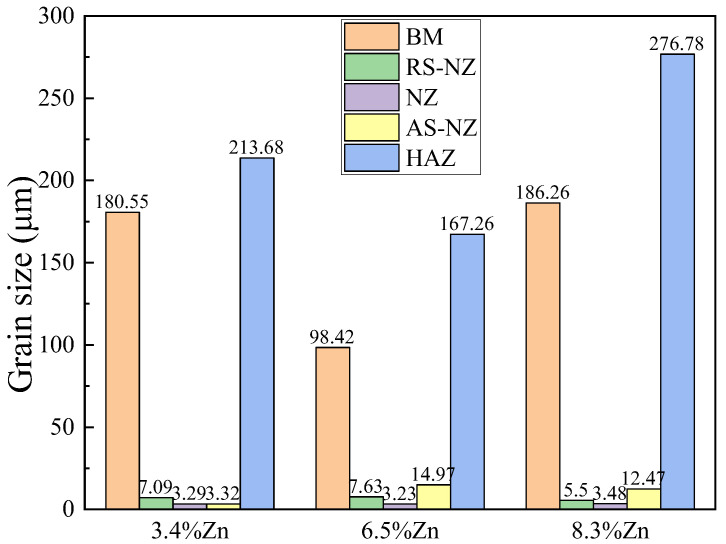
Grain size of different regions of FSWed joints.

From Figure 6b, Figure 7b, Figure 8b, Figure 10 and Figure 11, the grain size of α-Al of the as-cast Al-Si-Mg-Fe-6.5%Zn is the smallest (98.42 μm), followed by the as-cast Al-Si-Mg-Fe-3.4%Zn (180.55 μm), while the grain size of the α-Al of the as-cast Al-Si-Mg-Fe-8.3%Zn is the largest (186.26 μm). This indicates that with the increase in Zn content, the grain size of α-Al first decreases then increases. The as-cast Al-Si-Mg-Fe-6.5%Zn has most and the largest volume fractions of eutectic Al-Si and the percentage of Si in eutectic Al-Si increases (Figure 10b) due to the lowest melting temperature, resulting in the smallest grain size of α-Al. As mentioned earlier, Zn mainly dissolves in Al-Si-Mg-Fe-Zn and some precipitates (MgZn_2_) may form during casting. The difference in microstructure among the as-cast alloys is primarily related to the Zn content which affects the diffusion of Al and Si during the solidification process [78,79]. During eutectic solidification, since Zn was mainly enriched at the solid–liquid of the Al phase, hindering the growth of the a-Al, while the growth rate of the Si phase was higher [80]

**Figure 12:**
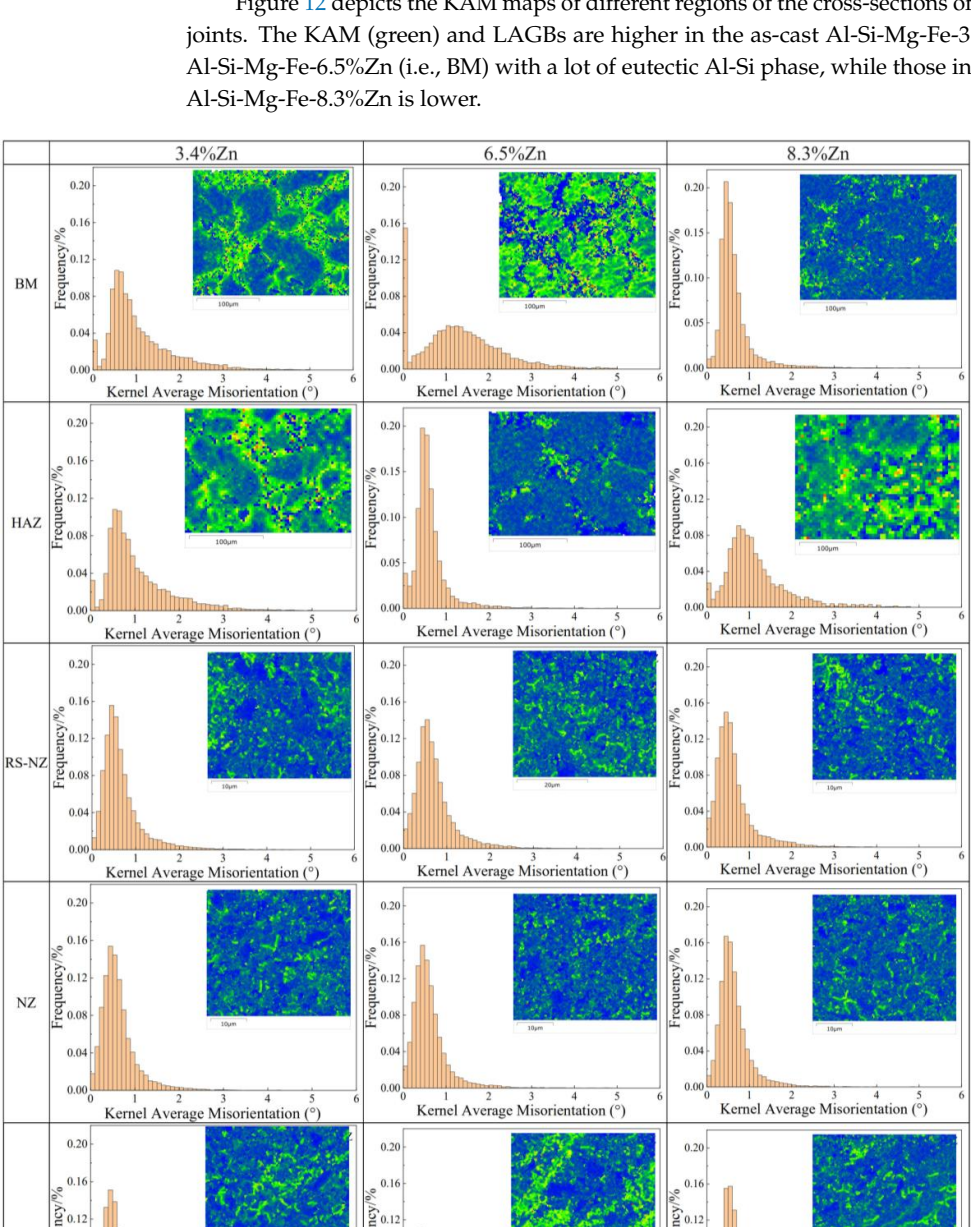
KAM maps of different zones of the cross-sections of FSWed joints.

Figure 12 depicts the KAM maps of different regions of the cross-sections of the FSWed joints. The KAM (green) and LAGBs are higher in the as-cast Al-Si-Mg-Fe-3.4%Zn and Al-Si-Mg-Fe-6.5%Zn (i.e., BM) with a lot of eutectic Al-Si phase, while those in the as-cast Al-Si-Mg-Fe-8.3%Zn is lower.

As the material flow [43,44], and temperature and microstructure at different regions of the FSWed specimens varies [41,42,81]. The welds show inherent asymmetry, with stronger plastic deformation in AS (Figure 6a,d,f, Figure 7a,d,f and Figure 8a,d,f). This phenomenon is caused by the velocity difference in the two sides, that is, higher velocity at AS while lower at RS, resulting in different heat effect fluidity and microstructure. During FSW, the rotating tool was inserted into a workpiece, and the plasticized material at the AS was moved to the RS and extruded, and then the material from RS was brought to AS. If the fluidity of the material is low, cavities will form at AS [82]. At the RS, the temperature distribution is more uniform [82], resulting in a gradual transition from NZ to TMAZ (Figure 6a, Figure 7a and Figure 8a). At the AS, the temperature gradient is relatively large, so the various zones at the AS is clearly observed.

Since the higher velocity at AS resulted in a larger strain rate and a higher temperature due to the larger degree of SPD and DRX of materials [83], the more heat energy is present at AS than the RS. Thus, the grain size of α-Al and % of LAGBs in the NZ at AS are larger than those in the RS of the NZ.

It is observed that the grain size in the HAZ is slightly coarser than that of the BM as shown in Figure 6c, Figure 7c, Figure 8c and Figure 11, mainly caused by only heat effect increasing the grain size. From Figure 11, the KAM of HAZ of the FSWed Al-Si-Mg-Fe-3.4%Zn and FSWed Al-Si-Mg-Fe-8.3%Zn is larger than that of BMs. While the KAM of the FSWed Al-Si-Mg-Fe-6.5%Zn is lower than that of BM.

**Figure 13:**
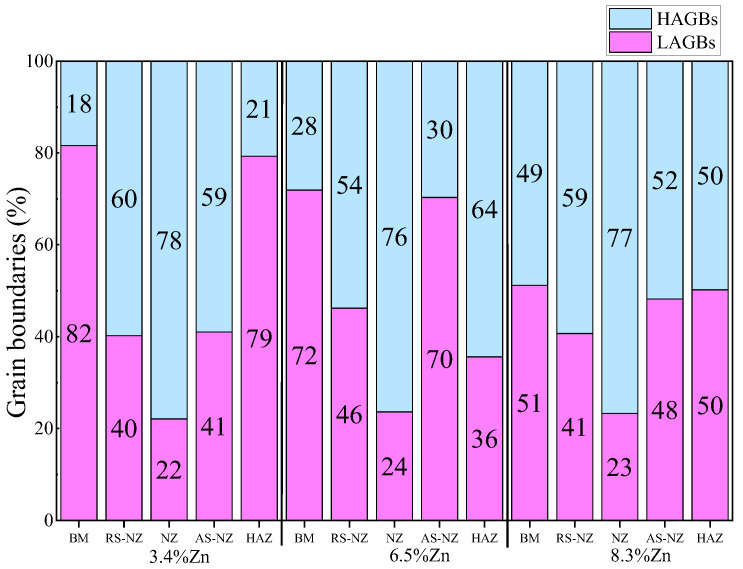
Percentage of LAGBs and HAGBs of different regions of FSWed joints.

The grains in AS-NZ and RS-NZ are refined (5.5 to 15 μm) and has a large degree of plastic deformation under the welding thermal cycle and mechanical stirring of the pin as shown in Figure 6d,f, Figure 7d,f, Figure 8d,f and Figure 11. The grains in AS-NZ and RS-NZ are larger than that in the NZ. The high-temperature thermal cycle and stirring of the pin could lead to SPD of the material which caused the DRX and the equiaxial fine grains in NZ [41,42,43,44,47,81,84,85,86]. From Figure 6e, Figure 7e and Figure 8e, fine equiaxed grains are observed in the NZ. It was found that the LAGBs in BM was changed into HAGBs in NZ under stirring of the pin and shoulder during FSW (Figure 13) [87]. Decrease in KAM shows that SPD occurs in the weld zone with the decrease in internal stress, since KAM is correlated with dislocation density [88]. The grain size of α-Al of the NZs of all FSWed joints is similar (3.23–3.48 μm). The % of LAGBs in NZs are 22–24% and much lower than that of the as-cast alloys (70–83%). The KAM and the % of LAGBs of the FSWed Al-Si-Mg-Fe-6.5%Zn are the highest, followed by FSWed Al-Si-Mg-Fe-8.3%Zn. This is mainly because the Al-Si eutectic phase is the most abundant in as-cast Al-Si-Mg-Fe-6.5%Zn. After FSW, Si was fragmented and stirred by the pin, and more dislocations are formed at the interface between Al and Si, thereby resulting in the increase in dislocation density.

**Figure 14:**
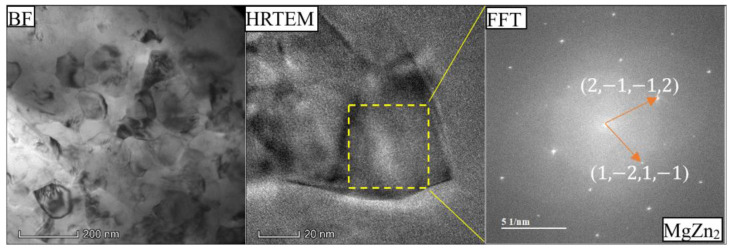
Bright-field (BF) TEM image of α-Al matrix, HRTEM image and Fast Fourier Transformation (FFT) of MgZn_2_ in as-cast Al-Si-Mg-Fe-8.3%Zn.

**Figure 15:**
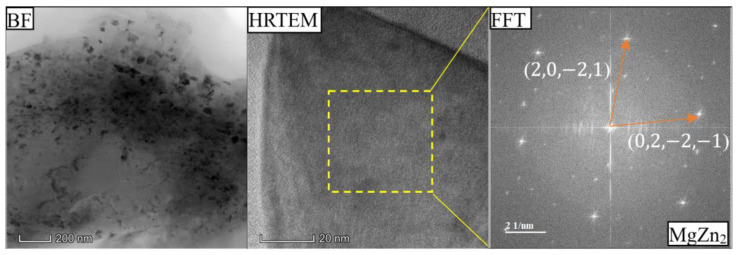
Bright-field (BF) TEM image of α-Al matrix, HRTEM image and Fast Fourier Transformation (FFT) of MgZn_2_ in FSWed Al-Si-Mg-Fe-8.3%Zn.

Figure 14 and Figure 15 show the bright-field (BF) TEM of the α-Al matrix, HRTEM image and Fast Fourier Transformation (FFT) of MgZn_2_ in the as-cast Al-Si-Mg-Fe-8.3%Zn and FSWed Al-Si-Mg-Fe-8.3%Zn. There are many black nano-precipitates in the α-Al matrix. The black and fine secondary phase, mainly MgZn_2_, is particularly abundant in the FSWed Al-Si-Mg-Fe-8.3%Zn, with size ranging from 20 to 40 nm (Figure 14), while the precipitates of MgZn_2_ in the as-cast Al-Si-Mg-Fe-8.3%Zn are larger (150 nm) as shown in Figure 13. Thus, there are many MgZn_2_ in Al-Si-Mg-Fe-8.3%Zn before and after FSW. The materials underwent SPD and DRX during FSW lead to grain refinement and strength enhancement in the joints.

Macroscopically, the FSWed joints without and with Zn are all well-formed and no defects can be seen on the surface (Figure 4) but no significant differences can be observed. The microstructures of NZ are similar, but there are some differences in grain size, number of secondary phases, internal stress, and texture, etc. These differences cause the variations in their mechanical and corrosion properties. The as-cast Al-Si-Mg-Fe-0%Zn possesses higher melting temperature (Figure 3) and less secondary phase, indicating that the higher temperature is required during FSW and the strength is lower. During FSW, Zn helps to refine the grains, but the refinement did not occur in a linear manner with an increase in Zn content. Meanwhile, Zn helps to slow down the decline of KAM and promote more changes in the shear texture.

### 3.3. XRD Study

**Figure 16:**
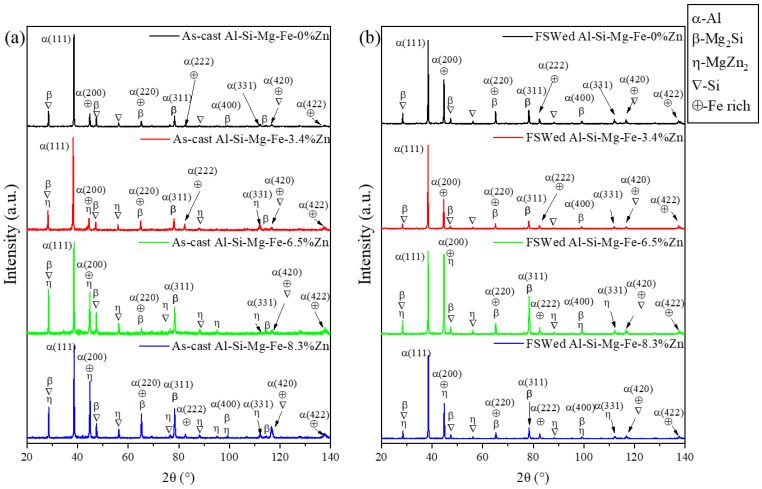
XRD patterns of (**a**) as-cast alloys and (**b**) FSWed joints with different Zn contents.

Figure 16 indicates XRD results of the as-cast alloys and the NZ of the FSWed joints. α-Al, Si, Fe-rich phase, Mg_2_Si (β) and MgZn_2_ (η) (absent in Al-Si-Mg-Fe-0%Zn) are detected in the as-cast alloys and the NZs of FSWed joints. It can be seen that the intensities of Mg-rich secondary phases (Mg_2_Si and MgZn_2_) decrease after FSW. Mg_2_Si and MgZn_2_ were dissolved in α-Al as the cooling rate is too fast, leading to fewer precipitates. But the precipitates are uniformly distributed and refine after FSW, as shown in Figure 14 and Figure 15 [47]. From the DSC results, there is almost no MgZn_2_ in FSWed Al-Si-Mg-Fe-6.5Zn, so the secondary phases is mainly the Si, Fe-rich phase, and Mg_2_Si.

### 3.4. XPS Study

**Figure 17:**
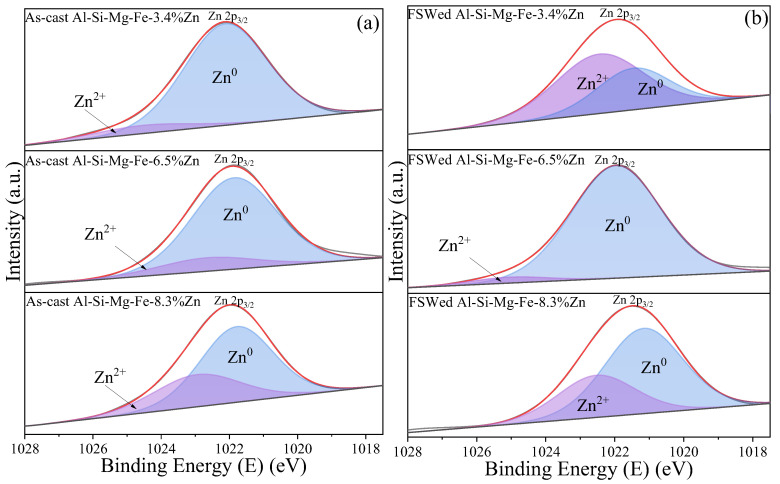
XPS narrow-scan spectra of Zn 2p for (**a**) as-cast alloys and (**b**) FSWed joints in NZ.

Figure 17 shows the narrow-scan XPS spectra of Zn 2p for different specimens. The Zn 2p_3/2_ binding energy for Zn^0^ is about 1021 eV and for Zn^2+^ is about 1022.5 eV [89]. The intensities of Zn^2+^ 2p for Al-Si-Mg-Fe-3.4%Zn and Al-Si-Mg-Fe-8.3%Zn increase after FSW, reflecting the increase in MgZn_2_. However, for Al-Si-Mg-Fe-6.5%Zn after FSW, the intensity decreases, indicating the decrease in MgZn_2_.

**Figure 18:**
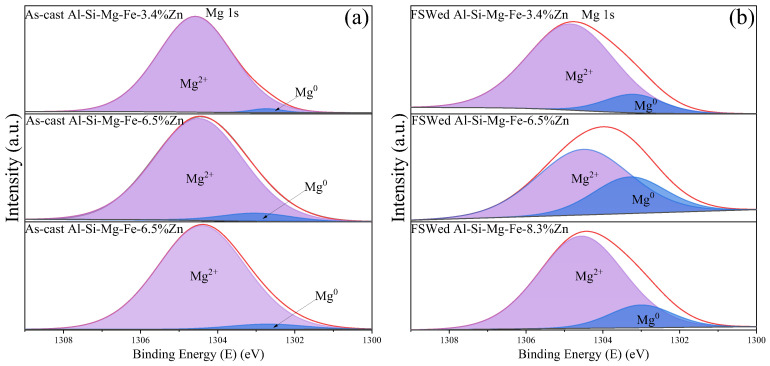
XPS narrow-scan spectra of Mg 1s for (**a**) the as-cast alloys and (**b**) the FSWed joints.

The narrow-scan XPS spectra of Mg 1s for different specimens are shown in Figure 18. The Mg 1s binding energies for Mg^0^ and Mg^2+^ are about 1033 eV and 1034.5 eV, respectively. In addition to Mg_2_Si, there is also Mg^2+^ in MgZn_2_. It can be noted that the peak shifts to the lower binding energy of Mg^0^ after FSW, especially for the Al-Si-Mg-Fe-6.5%Zn, indicating that the amount of Mg_2_Si decreases and the total amount of Mg_2_Si and MgZn_2_ also decreases.

Combining the results of DSC, XRD and XPS, the contents of Mg_2_Si and MgZn_2_ in the as-cast alloys increase with the increase in Zn. This is because more Zn can induce higher density of solute aggregates of Mg-Si and Mg-Zn [17,18,19]. After FSW, Si, Mg_2_Si and MgZn_2_ were dissolved in α-Al and the Fe-rich phases were fragmented and dispersed in α-Al, and some secondary phases were re-precipitated during cooling. The MgZn_2_ content in the NZs of the FSWed Al-Si-Mg-Fe-3.4%Zn and FSWed Al-Si-Mg-Fe-8.3%Zn slightly increases as compared with the as-cast alloys, but the MgZn_2_ content decreases in the FSWed Al-Si-Mg-Fe-6.5%Zn. As MgZn_2_ is completely dissolved into the α-Al matrix during FSW without precipitation during cooling. As the melting temperature of MgZn_2_ in Al-Si-Mg-Fe-6.5%Zn is the lowest, MgZn_2_ has the highest thermal sensitivity in Al-Si-Mg-Fe-6.5%Zn, leading to more MgZn_2_ dissolution during FSW. In addition, the Mg_2_Si content decreases in all specimens after FSW. Generally, the content of secondary phases decreases slightly.

### 3.5. Mechanical Properties

**Figure 19:**
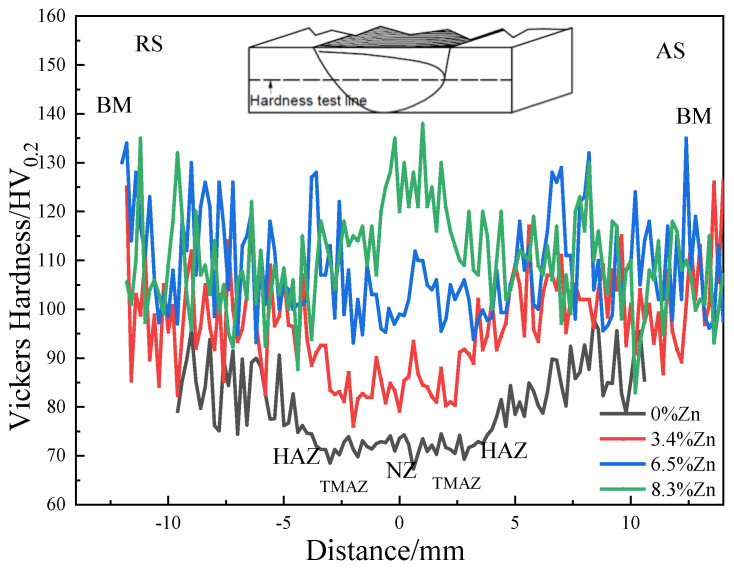
Hardness profiles across the cross-sections of FSWed joints.

Figure 19 reveals the hardness profiles of different FSWed joints. Figure 19 shows the stress–strain curves and Table 3 summarizes the extracted parameters, including the Young modulus (E), yield strength (YS), ultimate tensile strength (UTS), percent elongation (%EL), strength coefficient (the ratio of the UTS of the joint to the UTS of the as-cast alloy), and also the average hardness of the as-cast alloys and FSWed joints. The hardness, YS and UTS of the as-cast alloys increase first then slightly decrease with the increase in Zn content. There is a positive correlation between YS and hardness, the higher the yield strength, the greater the hardness [90].

There are mainly four ways to strengthen the alloys, outlined as follows: working hardening, solid solution strengthening, precipitation hardening, and grain size reduction [91]. The hardness is related to the grain size, and is governed by the Hall–Petch equation as follows [92,93,94]:

*H* = *H_o_* + 𝑘_𝑦_𝑑^−½^ (4)

where *H* is the hardness of specimen, *H_o_* is the whole resistance of dislocation movement to the lattice, *d* is the average grain size, and *k_y_* is the strengthening constant.

Thus, the highest hardness (110.3 ± 10.6 HV_0.2_), YS (181.1 ± 15.1 MPa) and UTS (206.1 ± 19.7 MPa) of the as-cast Al-Si-Mg-Fe-6.5%Zn are mainly attributed to the small grain size, the largest KAM (highest dislocation density), the highest amount of eutectic Al-Si phase [95,96], and relatively high contents of Mg_2_Si and MgZn_2_. The hardness (105.8 ± 7.1 HV_0.2_), YS (180.5 ± 14.6 MPa) and UTS (201.7 ± 18.2 MPa) of the as-cast Al-Si-Mg-Fe-8.3%Zn are slightly lower than those of the as-cast Al-Si-Mg-Fe-6.5%Zn, as there are more secondary phases although its KAM is the lowest.

From Figure 19, the hardness of the NZ of FSWed joints is slightly higher than that in TMAZ because the grains in the NZ are refined under the extrusion of the stirring pin and the shoulder, as well as uniform distribution of the reinforcements (Mg_2_Si and MgZn_2_). While the lower temperature at the HAZ and TMAZ leads to the accumulation of refined phases (similar to overaging) and thus reduces the strength during FSW [42].

The hardness profiles of the FSWed Al-Si-Mg-Fe-3.4%Zn and FSWed Al-Si-Mg-Fe-6.5%Zn are saddle-shaped (W shape). The hardness of the NZ is higher than those of the TMAZ and HAZ but lower than that of the as-cast alloys (BMs), which is consistent with lower strength for the FSWed joints than that of the as-cast alloys (Figure 19 and Table 3). This softening phenomenon of these two FSWed joints is mainly caused by the decrease in KAM (dislocation density), and the reduction in the amount of secondary phase (MgZn_2_) for the FSWed Al-Si-Mg-Fe-6.5%Zn.

In addition, the hardness of the NZ of the FSWed joint of Al-Si-Mg-Fe-8.3%Zn is the highest and gradually decreases from the center of the NZ. The hardness is reduced in TMAZ and HAZ, and close to that of the as-cast Al-Si-Mg-Fe-8.3%Zn, hence there is no reduction in hardness.

**Figure 20:**
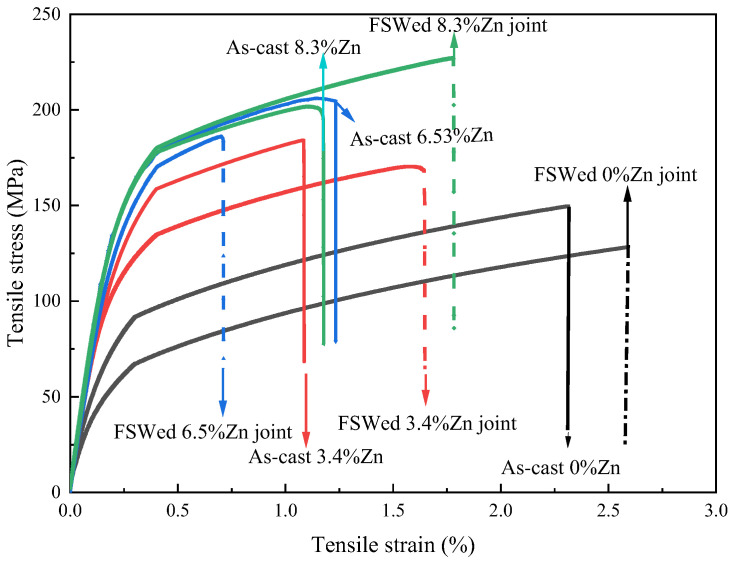
Tensile curves of as-cast alloys and FSWed joints.

From Figure 19 and Figure 20 and Table 3, the hardness, YS and UTS of the FSWed joints increase with the Zn content. The mechanical properties and strength coefficient of the FSWed Al-Si-Mg-Fe-8.3%Zn are the highest compared with the other two FSWed joints. Although KAM was much reduced, the strengthening of the FSWed Al-Si-Mg-Fe-8.3%Zn joint is mainly due to the grain refinement and the most secondary phases (Mg_2_Si and MgZn_2_) among the FSWed joints. Grain refinement and numerous and dispersed secondary phases can both impede dislocations, thereby enhancing mechanical properties. The Al-Si-Mg-Fe-8.3%Zn has a higher Zn content and generates more MgZn_2_. FSW involved the dissolution, precipitation and fragmentation of the secondary phase (undissolved). It was confirmed in the DSC results that the melting point of MgZn_2_ in the Al-Si-Mg-Fe-8.3%Zn was relatively high (i.e., more difficult to dissolve in the solid solution), and the amount of MgZn_2_ increased after FSW from the XRD result. Therefore, the precipitated MgZn_2_ is more than that dissolved into the α-Al matrix. Moreover, after FSW, the MgZn_2_ phase experienced SPD and turned into a large number of fine diffused phases. Meanwhile, Zn can reduce the vacancy concentration in the matrix, restrict the movement of vacancies [51,52], slow down the growth of the precipitates, and make the α-Al_12_(Fe, Mn)_3_Si more uniformly dispersed.

The FSWed Al-Si-Mg-Fe-6.5%Zn has the second highest hardness, YS and UTS, since though it has the least secondary phases (Mg_2_Si and MgZn_2_), it also has the highest KAM (most dislocation) and the smallest grain size.

Furthermore, the %EL of the FSWed Al-Si-Mg-Fe-8.3%Zn is the largest (1.78%), and the strength of the coefficient is over 100% indicating that the strength of the as-cast alloys can be maintained after FSW. The decrease in ductility of FSWed Al-Si-Mg-Fe-6.5%Zn is mainly due to the smallest grains, leading to restricted dislocation movement.

Moreover, the hardness, YS and UTS in the Al-Si-Mg-Fe-0%Zn are the lowest before and after FSW, since KAM is the lowest, there is no MgZn_2_ and less Mg_2_Si, and no Zn solid solution in the α-Al to impede dislocations (no lattice distortion caused by Zn). This means that during the tensile test, the ability to impede dislocations of as-cast and FSWed Al-Si-Mg-Fe-0%Zn is poor, and on the other hand, this is the reason for the good elongation.

**Figure 21:**
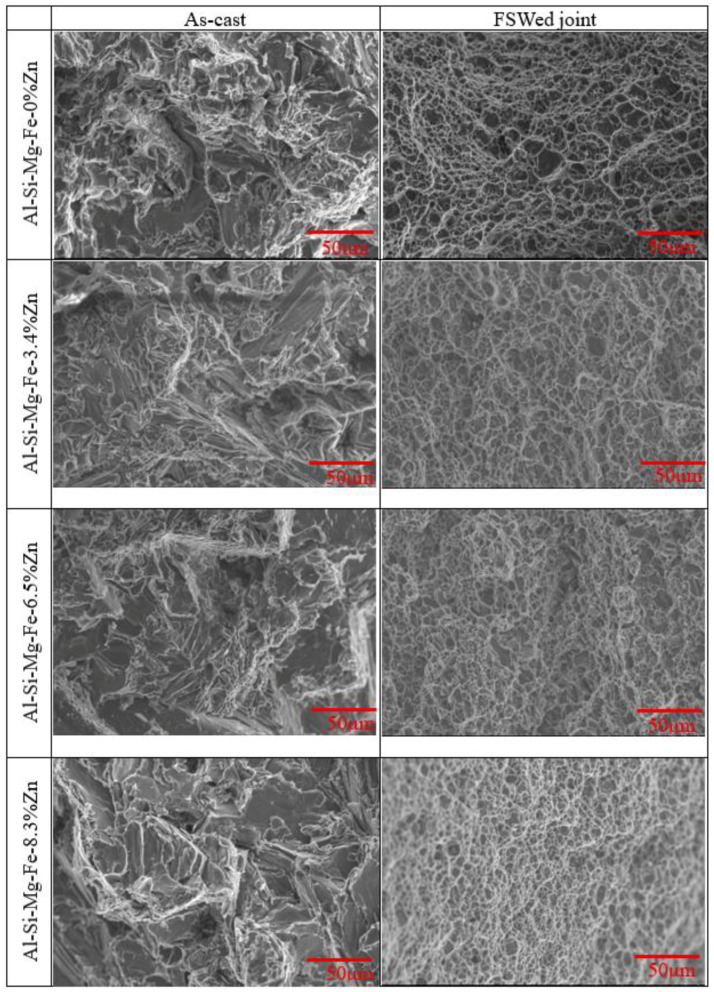
Fracture surface of various specimens after tensile test.

Figure 21 shows the fracture surfaces of the as-cast alloys and the FSWed joints. The fractured surface of the as-cast alloys shows both ductile and brittle fracture [97]. It can be seen that the fractured surface of the FSWed joints is dimple-like ductile fracture since the grains are refined. The dimple-like ductile fracture shows the good qualities of the joints [1]. The formation process of dimple-like ductile fracture in the FSWed joints is as follows: under the action of external force, due to strong slip, micro-voids are first formed mainly at the secondary phases and inclusions, and then these micro-voids undergo coalescence under the action of shear stress, and some new big voids produce resulting in the entire workpiece fracture [1]. The content of the secondary phases in the FSWed Al-Si-Mg-Fe-8.3%Zn is the highest, so the dimple-like ductile fracture surface is uniform and equiaxial, and the mechanical strength is the highest.

### 3.6. Corrosion Behavior

**Figure 22:**
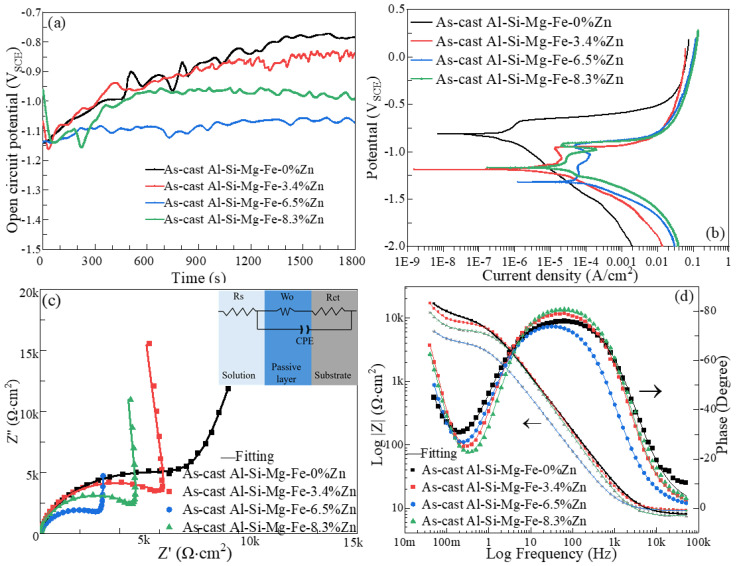
(**a**) OCP vs. time, (**b**) polarization curves, (**c**) Nyquist plots and (**d**) Bode plots of as-cast alloys.

**Figure 23:**
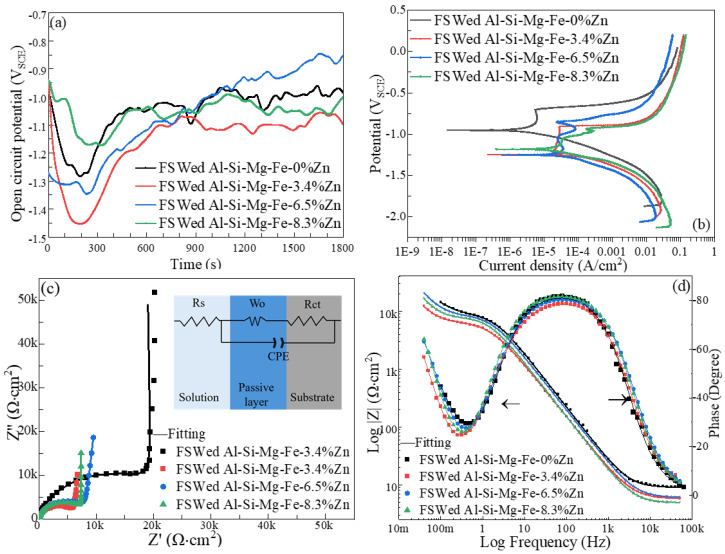
(**a**) OCP vs. time, (**b**) polarization curves, (**c**) Nyquist plots and (**d**) Bode plots of NZs of FSWed joints.

Figure 22 and Figure 23 show the plots of open circuit potential (OCP) vs. time, polarization curves, Nyquist and Bode plots of the as-cast alloys and the NZs of the FSWed joints in 3.5 wt% NaCl solution at 25 °C.

Table 4 summarizes the OCP, corrosion current density (I_corr_) and pitting potential (E_pit_) and Table 5 shows EIS fitting results of different specimens. The semi-circle is related to the constant-phase element CPE and charge transfer resistance *R_ct_* across the electrode interface, the diagonal line refers to the Warburg impedance *W*. *R_s_* is the solution resistance.

Overall, Al-Si-Mg-Fe-0%Zn with highest OCP, lowest I_corr_ and highest E_pit_ and largest R_CT_ has the best corrosion resistance (CR) before and after welding with highest. The following mainly discusses the samples containing Zn.

The OCP of the as-cast alloys and FSWed joints reached a stable state after 800 s and 1200 s, respectively. The OCP values of the FSWed joints decrease first then slightly increases with the increase in Zn content, and that of the FSWed Al-Si-Mg-Fe-6.5%Zn is the noblest mainly due to the least secondary phases such as MgZn_2_.

From Table 4 and Table 5, the as-cast Al-Si-Mg-Fe-3.4%Zn has the noblest OCP (−867 mV_SCE_)_,_ lowest I_corr_ (1.7 μA/cm^2^) and highest R_ct_ (9245.16 Ω·cm^2^), indicating the highest CR. The as-cast Al-Si-Mg-Fe-6.5%Zn possesses the most active OCP (−1071 mV_SCE_), the largest I_corr_ (35.4 μA/cm^2^), and the lowest R_ct_ (4663.92 Ω·cm^2^), reflecting the lowest CR. The CR of the as-cast Al-Si-Mg-Fe-8.3%Zn is in the middle with OCP of −971 mV_SCE_, I_corr_ of 8.3 μA/cm^2^, and R_ct_ of 6934.04 Ω·cm^2^. In addition, the E_pit_ increases with the Zn content first but then slightly decreases. E_pit_ mainly reflects the sensitivity to the occurrence of corrosion pits. The as-cast Al-Si-Mg-Fe-3.4%Zn has the lowest E_pit_ due to the larger Fe-rich phases as shown in Figure 10 and Appendix A, which provide more active sites for pitting attack. The E_pit_ of the as-cast Al-Si-Mg-Fe-6.5%Zn and as-cast Al-Si-Mg-Fe-8.3%Zn are higher due to the smaller Fe-rich phases.

**Figure 24:**
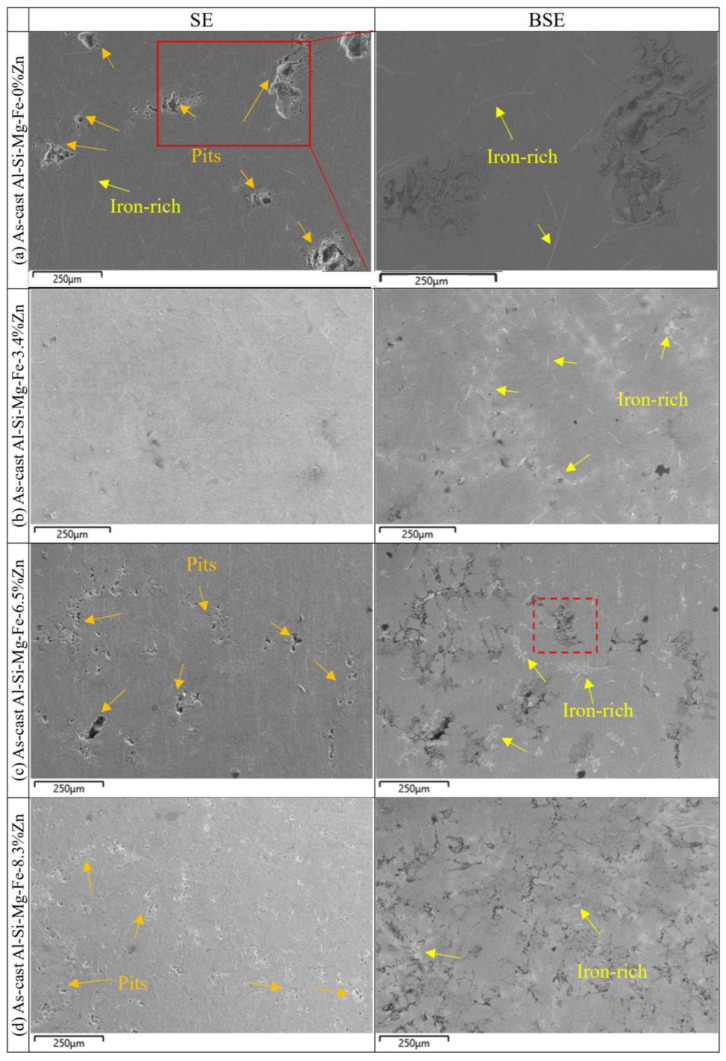
SE and BSE images of the corroded surface of as-cast alloys after polarization test.

The FSWed Al-Si-Mg-Fe-6.5%Zn possesses the noblest OCP (−898 mV_SCE_), highest E_pit_ (−849 mV_SCE_), lowest I_corr_ (10.4 μA/cm^2^) and highest R_ct_ (9192.37 Ω·cm^2^) among the FSWed joints. The FSWed joint of Al-Si-Mg-Fe-3.4%Zn has the lowest OCP (1094 mV_SCE_), largest I_corr_ (31 μA/cm^2^), and lowest R_ct_ (6840.83 Ω·cm^2^), i.e., the lowest CR. The CR of the FSWed joint of Al-Si-Mg-Fe-8.3%Zn is in the middle with E_OCP_ (1044 mV_SCE_), I_corr_ (20.9 μA/cm^2^), and R_ct_ (8413.83 Ω·cm^2^). From the Bode diagram that the low-frequency impedance modulus (the |Z| of FSWed Al-Si-Mg-Fe-6.5%Zn > FSWed Al-Si-Mg-Fe-8.3%Zn > FSWed Al-Si-Mg-Fe-3.4%Zn) also corresponds to the corrosion behavior (the CR of FSWed Al-Si-Mg-Fe-6.5%Zn > FSWed Al-Si-Mg-Fe-8.3%Zn > FSWed Al-Si-Mg-Fe-3.4%Zn). From Figure 24, the FSWed Al-Si-Mg-Fe-6.5%Zn is mildly corroded.

**Figure 25:**
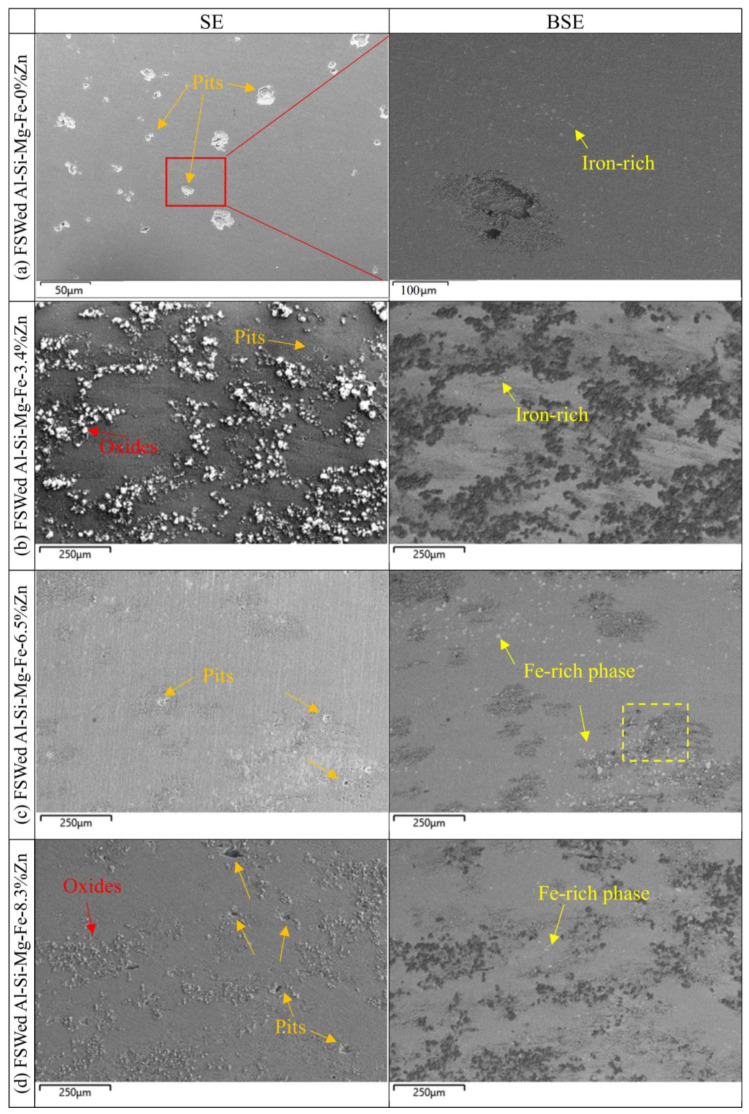
SE and BSE images of the corroded surface of NZs of FSWed joints after polarization test.

Figure 24 and Figure 25 show SE and BSE images of the corrosion morphology of the as-cast alloys and the NZ of the FSWed joints, respectively. The CR of the as-cast and FSWedAl-Si-Mg-Fe-0%Zn is the highest before and after welding because there is fewer secondary phases (no MgZn_2_, micro-galvanic effect reduced) and the smallest KAM (lower internal energy), the corrosion resistance is the best in Al-Si-Mg-Fe-0%Zn. However, there is still severe local corrosion in the as-cast and FSWed Al-Si-Mg-Fe-0%Zn, although it can be seen from the polarization curve and the maximum R_ct_ that the corrosion performance of Al-Si-Mg-Fe-0%Zn is the best. It was found in the EIS results that the value of W-R was the smallest, indicating that the local passivation film was thin.

From the BSE images (Figure 24 and Figure 25), the brightest regions are mainly the Fe-rich phase as the atomic mass of Fe is the highest. From Figure 24, mild corrosion is observed in the as-cast Al-Si-Mg-Fe-3.4%Zn, there are many corrosion products and some small pits in the as-cast Al-Si-Mg-Fe-8.3%Zn, while the as-cast Al-Si-Mg-Fe-6.5%Zn shows severe pitting corrosion. Thus, the corrosion resistance of the as-cast Al-Si-Mg-Fe-3.4%Zn is the highest, followed by that of as-cast Al-Si-Mg-Fe-8.3%Zn, and that of the as-cast Al-Si-Mg-Fe-6.5%Zn is the worst. The pits are found at the α-Al (more anodic) near the Fe-rich phases (cathodic) [98] due to micro-galvanic effect between them.

From Figure 25, the FSWed Al-Si-Mg-Fe-6.5%Zn is mildly corroded, and the FSWed Al-Si-Mg-Fe-8.3%Zn has some oxides and small pits, while that of the FSWed joint of Al-Si-Mg-Fe-3.4%Zn shows more corrosion products and small pits. For the FSWed Al-Si-Mg-Fe-3.4%Zn, the corroded surface has many corrosion products, and the corrosion resistance is apparently worse than the as-cast Al-Si-Mg-Fe-3.4%Zn and the other two FSWed joints. For the FSWed Al-Si-Mg-Fe-6.5%Zn and FSWed Al-Si-Mg-Fe-8.3%Zn, the corroded surface has less big pits than the as-cast Al-Si-Mg-Fe-6.5%Zn and as-cast Al-Si-Mg-Fe-8.3%Zn.

**Figure 26:**
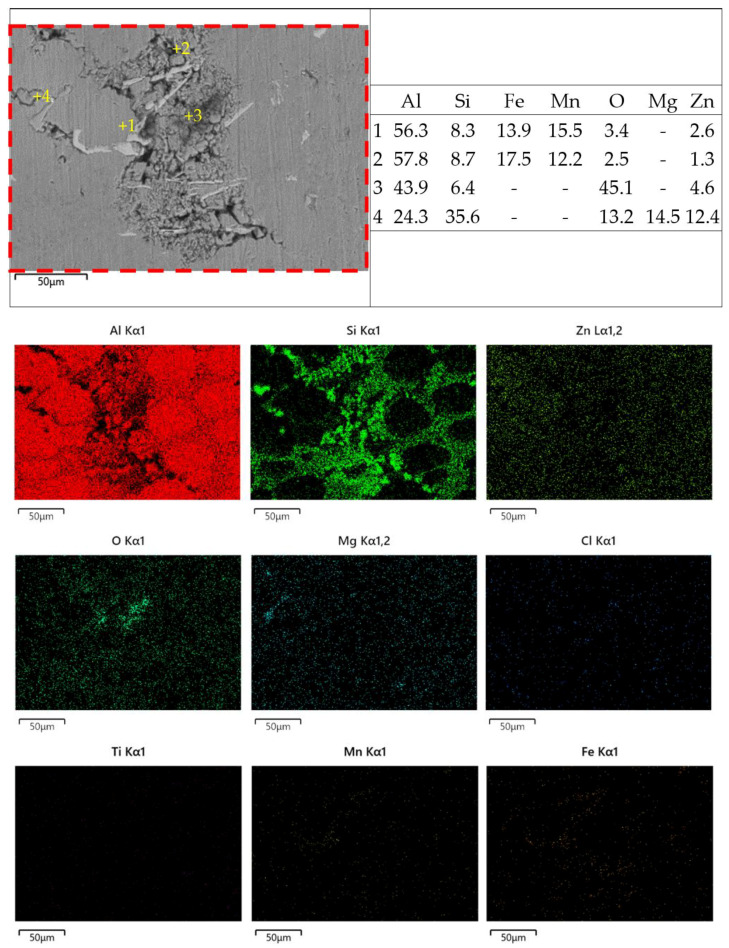
High magnification BSE image, the elements of some points and the EDS mapping of surface corrosion morphology of as-cast Al-Si-Mg-Fe-6.5%Zn.

**Figure 27:**
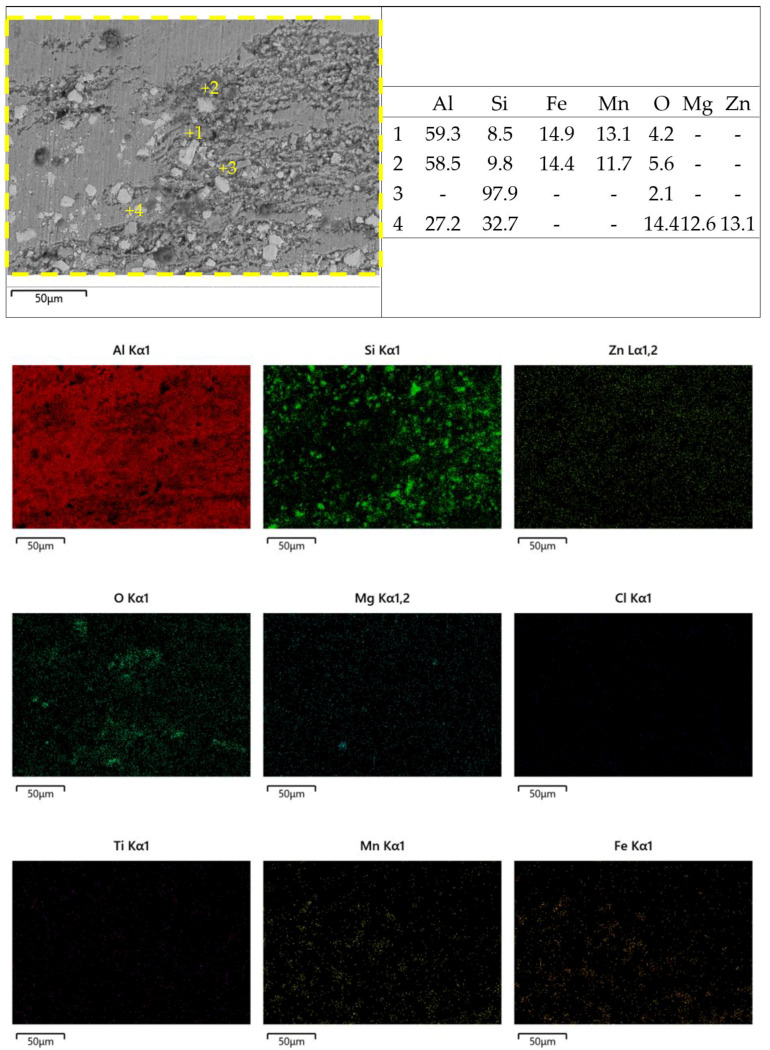
High magnification BSE image, the elements of some points and the EDS mapping of surface corrosion morphology of FSWed joint of Al-Si-Mg-Fe-6.5%Zn.

Figure 26 and Figure 27 show the zoom-in BSE images (from Figure 22b and Figure 23b) with EDX maps of the as-cast Al-Si-Mg-Fe-6.5%Zn and FSWed Al-Si-Mg-Fe-6.5%Zn, respectively. Many phases are detected in the as-cast alloys and the FSWed joints, which induced micro-galvanic effect among them in 3.5% NaCl solution [99]. Figure 27 shows the schematic diagram of the corrosion mechanism and illustrates the corrosion process of the Al-Si-Mg-Fe-Zn alloys with various phases, including α-Al, Si, Mg_2_Si, MgZn_2_, and Fe-rich phase in 3.5% NaCl solution.

**Figure 28:**
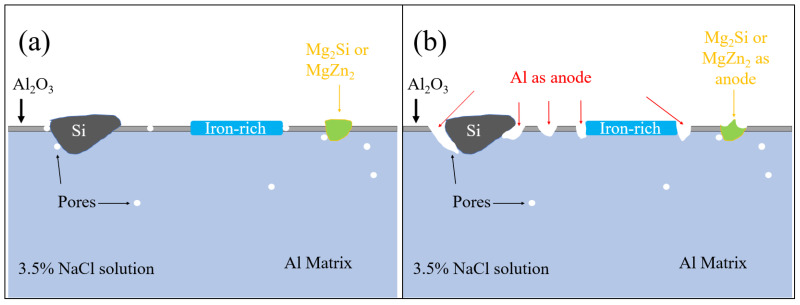
Corrosion mechanism of Al-Si-Mg-Fe-Zn alloys (**a**) before and (**b**) after corrosion test.

From Figure 28, these secondary phases have different electrochemical potentials against the α-Al matrix, leading to the induction of a micro-galvanic effect. The ranking of corrosion potentials of various phases are the following [100]:

Fe-rich phase > Si > α-Al > Mg_2_Si > MgZn_2_

The reactions are as follows [101,102,103,104]:

Anodic reaction: Al → Al^3+^ + 3e^−^ (5)

Anodic reaction: MgZn_2_ → Mg^2+^ +2Zn^2+^ + 6e^−^ (6)

Anodic reaction: Mg_2_Si + 2H_2_O → Mg^2+^ + SiO_2_ +4H^+^ + 8e^−^ (7)

Cathodic reaction: O_2_ + 2H_2_O + 4e^−^ → 4OH^−^ (8)

Therefore, the micro-galvanic effect was significant during the corrosion tests. The rapid corrosion began at MgZn_2_ and then Mg_2_Si [100,105]. From Figure 26 and Figure 27, the small anodic Mg-rich phases were seriously corroded. The Mg-rich phases were preferentially dissolved first, and the α-Al matrix remained. The dissolution of MgZn_2_ generates Mg^2+^ and Zn^2+^ ions. Since the reduction potentials of Zn^2+^ and Mg^2+^ is more positive than that of Al^3+^, Zn^2+^ and Mg^2+^ are reduced through the reaction (Zn^2+^/Mg^2+^ + Al → Al^3+^ + Zn/Mg) and deposited near the pit. The oxide film was deteriorated, and the pit was enlarged rapidly [106,107]. In addition, when Mg_2_Si was dissolved, and Si is enriched in the alloys, and finally the Mg_2_Si shows cathode activity [108]. As the contents of MgZn_2_ and Mg_2_Si were low, the cathode-to-anode ratio is high owing to the small anodes (MgZn_2_ and Mg_2_Si) and large cathode (α-Al), leading to selective attack of the more active phases (MgZn_2_ and Mg_2_Si).

After MgZn_2_ and Mg_2_Si were completely corroded, the α-Al phases (large area) also corroded as the anodic site around the nobler phases (Si in eutectic Al-Si, and Fe-rich phase). So, corrosion all occurred in the α-Al around these nobler phases. When the α-Al around the secondary phases was severely corroded, the secondary phases fell off the alloy. It can be observed from Figure 26 and Figure 27 that the severely corroded areas are at the Al-Si eutectic in the as-cast alloys and the dispersed Al-Si eutectic in FSW joints.

Furthermore, pitting corrosion easily occurred in the pores and the secondary phases [109,110]. The secondary phases are active sites for pitting attack while the pores caused by casting are prone to oxygen deficiency and it is difficult to form an oxide film for protection. The corrosion behavior of the aluminum alloys is affected by many factors, including precipitates, grain boundary and size, compositions of the solid solution, and defects [111]. In addition, the KAM also affects corrosion behavior. The lower KAM means lower dislocation density, lower internal energy and macro-strain, resulting in fewer active sites for pitting corrosion [112]. From the previous results, the CR of the as-cast Al-Si-Mg-Fe-3.4%Zn is the highest, caused by the fewest coarse and coalescence secondary phases such as the original Mg_2_Si and MgZn_2_, though its KAM and grain size are large. The as-cast Al-Si-Mg-Fe-8.3%Zn less corrosion resistant than the as-cast Al-Si-Mg-Fe-3.4%Zn, owing to the lowest KAM, the fewest eutectic Al/Si phases, though the grain size is the largest and the content of the secondary phases is the highest. The as-cast Al-Si-Mg-Fe-6.5%Zn has the lowest CR, mainly resulting from the highest KAM and LAGBs, the more Si inducing stronger micro-galvanic effect (the surrounding Al as anode) and some coarse secondary phases like original iron-rich, Mg_2_Si and MgZn_2_.

After FSW, the contents of Mg_2_Si and MgZn_2_ decrease and are scattered in the α-Al matrix, which can increase the CR also as the protective layer (they corroded first). Furthermore, lower internal energy indicates the fewer active areas and lower dislocation density and less micro-strain for pitting corrosion, leading to higher CR [112]. Furthermore, smaller grain size also can cause higher CR [113,114], as it can increase the grain boundary area which can absorb the misorientation [115,116,117]. The advantage of the grain boundaries is for forming stable and dense passive film, whereas the disadvantage is that grain boundaries provide a large number of active dissolution sites [118]. The high-density and close-packed crystallographic planes have higher CR, as they facilitate the formation of passive film [119,120,121]. The most densely packed crystallographic plane in the FCC α-Al is the close-packed {111} plane. After FSW, simple shear textures increase representing a larger percentage of close-packed {111} plane. As reported in the previous work [98], the pores in the as-cast Al-Si-Mg-Fe-Zn were eliminated after FSW, and the CR was enhanced.

There are many factors that contribute to the highest CR of FSWed Al-Si-Mg-Fe-6.5%Zn. The grain size is the smallest, KAM is low, no MgZn_2_, less secondary phases and most percentage of {111} plane in NZ of FSWed Al-Si-Mg-Fe-6.5%Zn. The CR of the NZ of the FSWed Al-Si-Mg-Fe-8.3%Zn is also higher than as-cast Al-Si-Mg-Fe-8.3%Zn and just slightly lower than the NZ of the FSWed Al-Si-Mg-Fe-6.5%Zn joint, which is mainly caused by the refined grains, decease of KAM, and the fewer and smaller dispersed secondary phases and much close-packed {111} plane after FSW but the existence of many MgZn_2_ resulted in lower CR. Compared with the CR of the NZ of the FSWed Al-Si-Mg-Fe-6.5%Zn and FSWed Al-Si-Mg-Fe-8.3%Zn, that of FSWed Al-Si-Mg-Fe-3.4%Zn is the lowest, mainly resulting from the least close-packed {111} plane, higher KAM and some MgZn_2_. In addition, the CR of the FSWed Al-Si-Mg-Fe-3.4%Zn is worse than the as-cast Al-Si-Mg-Fe-3.4%Zn due to segregation of Zn at the grain boundaries for the FSWed Al-Si-Mg-Fe-3.4%Zn, leading to deterioration of CR and offsetting the benefits brought by FSW, including grain refinement and low KAM [122].

## 4. Conclusions

Zn significantly modifies the weld microstructure by acting as a grain refiner, promoting a finer grain structure in the NZ, and influencing precipitate formation [55,56]. With the addition of Zn, more strengthening precipitates MgZn_2_ and Mg_2_Si are formed, and their size, distribution, and stability are affected. Furthermore, Zn interacts with other elements like Mg, Fe, and Si to modify the morphology and distribution of intermetallic compounds (IMCs), potentially mitigating their detrimental effects on mechanical properties. The addition of Zn consequently enhances the mechanical properties of FSWed joints; it increases strength and hardness through solid solution strengthening and precipitation hardening, and also influences fractured behavior, potentially improving ductility. The strengthening precipitate MgZn_2_ can coarsen or dissolve in the α-Al matrix under the high welding temperatures, potentially forming softened areas; however, subsequent SPD and DRX during FSW, followed by air cooling, often lead to refined and dispersed MgZn_2_ precipitates, ultimately increasing strength. Simultaneously, dissolved Zn atoms in the α-Al matrix create strain fields that hinder dislocation movement, further enhancing the weld strength. Additionally, lower melting point of Zn compared to Al promotes the formation of low-melting-point eutectic structures at interfaces, improving weldability. Finally, Zn can influence material flow during FSW, potentially resulting in a more uniform and defect-free weld. The following conclusion is about the Al alloys with Zn contents.

- As the Zn content increases, the melting temperature of Al-Si-Mg-Fe-Zn alloys decreases and the contents of Mg_2_Si and Mg_2_Zn increase since Zn induces their aggregation. Moreover, Fe-rich phases are finer, more evenly distributed since the addition of Zn can promote the transformation of Al_6_(Fe, Mn) to finer, more dispersed, and more evenly distributed α-Al_12_(Fe, Mn)_3_Si. After FSW, the MgZn_2_ easily dissolved in Al-Si-Mg-Fe-6.5%Zn due to its lowest melting temperature.
- The hardness, YS and UTS of the as-cast alloys increase a lot first then slightly decrease with the Zn content. The largest hardness (110.3 HV_0.2_), YS (181.1 MPa) and UTS (206.1 MPa) in the as-cast Al-Si-Mg-Fe-6.5%Zn mainly results from the smallest grain size, the largest KAM (dislocation density), the highest content of eutectic Al/Si phase, and many Mg_2_Si and MgZn_2_. While the hardness (105.8 HV_0.2_), YS (180.45 MPa) and UTS (201.65 MPa) in as-cast Al-Si-Mg-Fe-8.3%Zn is slightly lower.
- The softening phenomenon in the weld zone of the alloys with 3.4%Zn and 6.5%Zn is mainly due to the reduction in KAM and fewer secondary phases (Si and MgZn_2_). The weld zone of 8.3%Zn is harder than the base material, and its mechanical properties with hardness (116.31 HV_0.2_), YS (184.37 MPa) and UTS (226.93 MPa) are the highest, mainly due to refined grains and the more diffused strengthening secondary phase like MgZn_2_, and largest EL% (1.78%), and strength coefficient over 100% indicating that the joint can maintain the strength of the as-cast alloy.
- The corrosion resistance of the as-cast Al-Si-Mg-Fe-3.4%Zn is the highest, caused by the fewest coarse and coalesce Mg_2_Si and MgZn_2_. The corrosion resistance of the as-cast Al-Si-Mg-Fe-8.3%Zn is the second one owing to the lowest KAM and least eutectic Al-Si. The corrosion resistance of the as-cast Al-Si-Mg-Fe-6.5%Zn is the lowest, attributed to the highest KAM and LAGBs, the most Si and many coarse and coalesce Mg_2_Si and MgZn_2_.
- The highest corrosion resistance of FSWed Al-Si-Mg-Fe-6.5%Zn is due to the smallest grain size and KAM, less secondary phases without MgZn_2_ and most {111} plane in NZ of FSWed 6.5%Zn joint. The corrosion resistance of FSWed Al-Si-Mg-Fe-8.3%Zn is also higher than the as-cast Al-Si-Mg-Fe-8.3%Zn and just slightly lower than FSWed Al-Si-Mg-Fe-6.5%Zn, which mainly caused by the refined grain size, decease of KAM, and the dispersed secondary phases and more close-packed {111} plane but the existence of many MgZn_2_ reduces its corrosion resistance.

## Figures and Tables

**Table 1:** Chemical compositions (wt%) of different Al-Si-Mg-Fe-Zn alloys.

Specimen	Al	Si	Zn	Mg	Fe	Mn	Ti	Sr
Al-Si-Mg-Fe-0%Zn	Bal.	10.1	0.0	0.5	0.6	0.4	0.1	0.02
Al-Si-Mg-Fe-3.4%Zn	Bal.	10.4	3.4	0.5	0.6	0.4	0.2	0.04
Al-Si-Mg-Fe-6.5%Zn	Bal.	10.2	6.5	0.5	0.6	0.4	0.1	0.02
Al-Si-Mg-Fe-8.3%Zn	Bal.	9.8	8.3	0.5	0.6	0.4	0.1	0.01

**Table 2:** DSC results of different specimens.

Specimen	α-Al Endothermic Peak		MgZn_2_ Endothermic Peak	
	Melting Enthalpy (J/g)	T_peak_ (°C)	Melting Enthalpy (J/g)	T_peak_ (°C)
As-cast Al-Si-Mg-Fe-0%Zn	590.8	581.5	-	-
As-cast Al-Si-Mg-Fe-3.4%Zn	524.5	576.8	3.09	542.5
As-cast Al-Si-Mg-Fe-6.5%Zn	459.5	568.5	4.13	517.3
As-cast Al-Si-Mg-Fe-8.3%Zn	437.9	567.1	8.14	530.8
FSWed Al-Si-Mg-Fe-0%Zn	661.5	579.6	-	-
FSWed Al-Si-Mg-Fe-3.4%Zn	534.0	576.9	576.9	544.5
FSWed Al-Si-Mg-Fe-6.5%Zn	423.2	571.9	-	-
FSWed Al-Si-Mg-Fe-8.3%Zn	497.4	568.2	8.21	534.6

**Table 3:** Mechanical parameters and average hardness of various specimens.

Specimen	E (GPa)	YS (MPa)	UTS (MPa)	%EL (%)	Strength Coefficient (%)	Average Hardness (HV_0.2_)
As-cast Al-Si-Mg-Fe-0%Zn	30.9 ± 2.7	99.8 ± 12.3	169.0 ± 23.7	2.3	-	86.3 ± 6.2
As-cast Al-Si-Mg-Fe-3.4%Zn	46.8 ± 3.4	163.4 ± 12.2	184.1 ± 15.2	1.1	-	98.0 ± 11.3
As-cast Al-Si-Mg-Fe-6.5%Zn	59.7 ± 5.2	181.1 ± 15.1	206.1 ± 19.7	1.2	-	110.3 ± 10.6
As-cast Al-Si-Mg-Fe-8.3%Zn	57.4 ± 3.8	180.5 ± 14.6	201.7 ± 18.2	1.2	-	105.8 ± 7.1
FSWed Al-Si-Mg-Fe-0%Zn	20.8 ± 2.3	76.2 ± 10.2	153.8 ± 20.5	2.6	91.0	71.8 ± 1.8
FSWed Al-Si-Mg-Fe-3.4%Zn	45.2 ± 3.5	137.6 ± 12.5	170.5 ± 13.2	1.7	92.6	86.7 ± 5.8
FSWed Al-Si-Mg-Fe-6.5%Zn	52.5 ± 4.2	176.5 ± 14.2	185.9 ± 16.4	0.7	90.1	103.6 ± 8.8
FSWed Al-Si-Mg-Fe-8.3%Zn	58.5 ± 5.3	184.4 ± 17.5	226.9 ± 20.3	1.8	112.5	116.3 ± 9.0

**Table 4:** Corrosion parameters of as-cast alloys and NZ of FSWed joints.

Specimen	OCP (mV_SCE_)	I_corr_ (μA/cm^2^)	E_pit_ (mV_SCE_)
As-cast Al-Si-Mg-Fe-0%Zn	−787 ± 22	0.4 ± 0.1	−740.5 ± 28
As-cast Al-Si-Mg-Fe-3.4%Zn	−867 ± 15	1.7 ± 1.1	−955 ± 10
As-cast Al-Si-Mg-Fe-6.5%Zn	−1071 ± 13	35.4 ± 5.2	−937 ± 13
As-cast Al-Si-Mg-Fe-8.3%Zn	−971 ± 17	8.3 ± 3.5	−941 ± 15
FSWed Al-Si-Mg-Fe-0%Zn	−994 ± 30	2.9 ± 1.4	−707 ± 26
FSWed Al-Si-Mg-Fe-3.4%Zn	−1094 ± 24	31.0 ± 8.3	−904 ± 12
FSWed Al-Si-Mg-Fe-6.5%Zn	−898 ± 20	10.4 ± 4.0	−849 ± 7
FSWed Al-Si-Mg-Fe-8.3%Zn	−1044 ± 23	20.9 ± 5.2	−928 ± 12

**Table 5:** EIS fitting results of as-cast alloys and NZ of FSWed joints.

Specimen	χ^2^ × 10^−3^	*R_s_* (Ω·cm^2^)	*R_ct_* (Ω·cm^2^)	W-*R* (Ω·cm^2^)	W-*Y*_0_ (SS^n^ cm^−2^ × 10^−3^)	W-*N* (SS^n^cm^−2^)	CPE-*Y*_0_ (SS^n^ cm^−2^ × 10^−6^)	CPE-*N* (SS^n^cm^−2^)
As-cast Al-Si-Mg-Fe-0%Zn	0.95	7.91 ± 0.05	11,375 ± 234	19	2	0.42	2.17	0.87
As-cast Al-Si-Mg-Fe-3.4%Zn	0.47	9.27 ± 0.03	9245 ± 426	559	167	0.53	8.90	0.92
As-cast Al-Si-Mg-Fe-6.5%Zn	2.5 × 10^−7^	9.14 ± 0.18	4627 ± 1	37	24	0.50	46.18	0.87
As-cast Al-Si-Mg-Fe-8.3%Zn	0.12	7.48 ± 0.05	6934 ± 80	8116	298	0.50	18.03	0.93
FSWed Al-Si-Mg-Fe-0%Zn	5.2	9.36 ± 0.10	9546 ± 300	11	1	0.43	1.07	0.88
FSWed Al-Si-Mg-Fe-3.4%Zn	0.31	5.71 ± 0.02	6167 ± 73	2184	770	0.48	18.88	0.91
FSWed Al-Si-Mg-Fe-6.5%Zn	0.78	6.00 ± 0.07	9192 ± 155	15,557	333	0.47	14.23	0.92
FSWed Al-Si-Mg-Fe-8.3%Zn	1.02	4.87 ± 0.05	8742 ± 110	13,149	352	0.51	1.07	0.94

**Figure A2:**
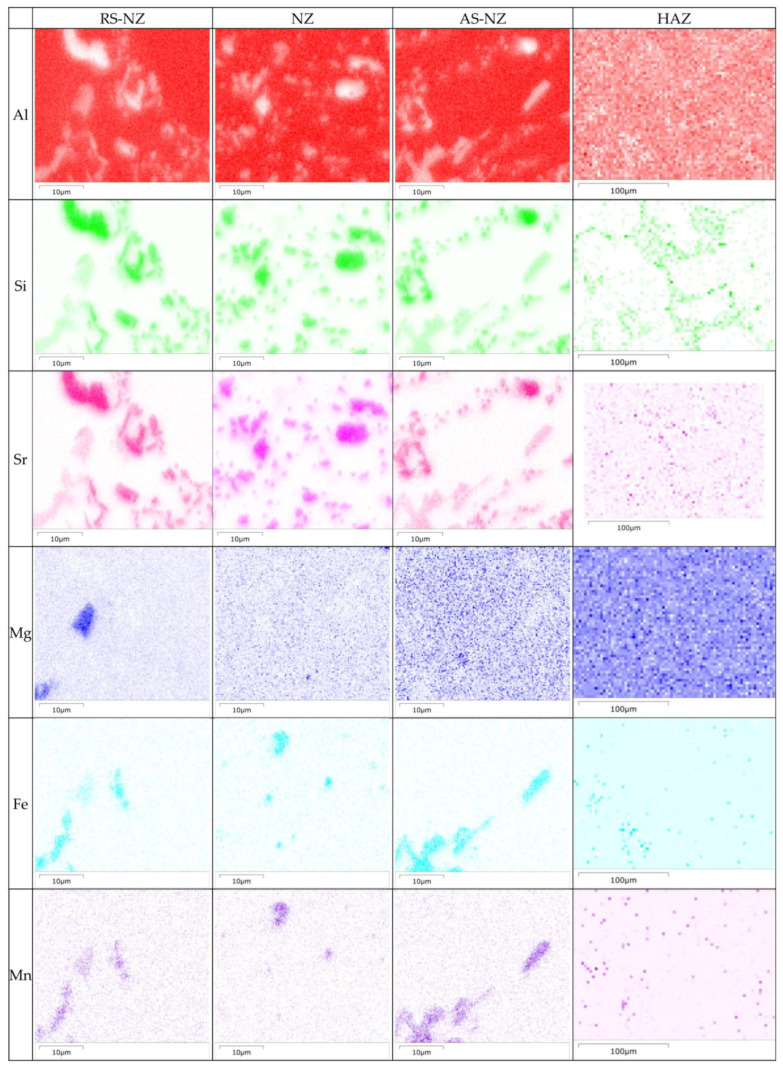

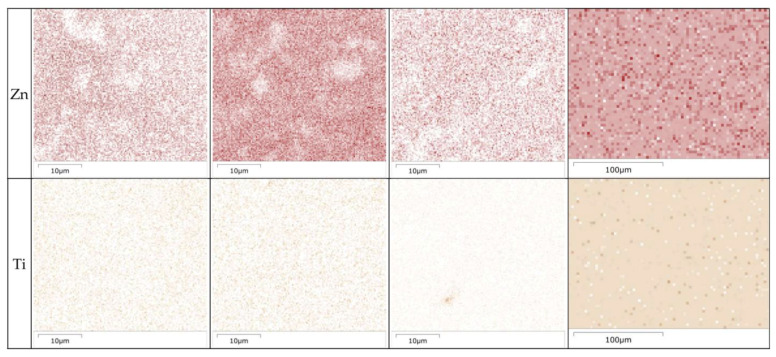
EDS maps of Al, Si, Sr, Mg, Fe, Zn and Ti of different regions of cross-sectional microstructures of the FSWed joint of Al-Si-Mg-Fe-3.4%Zn.

**Figure A3:**
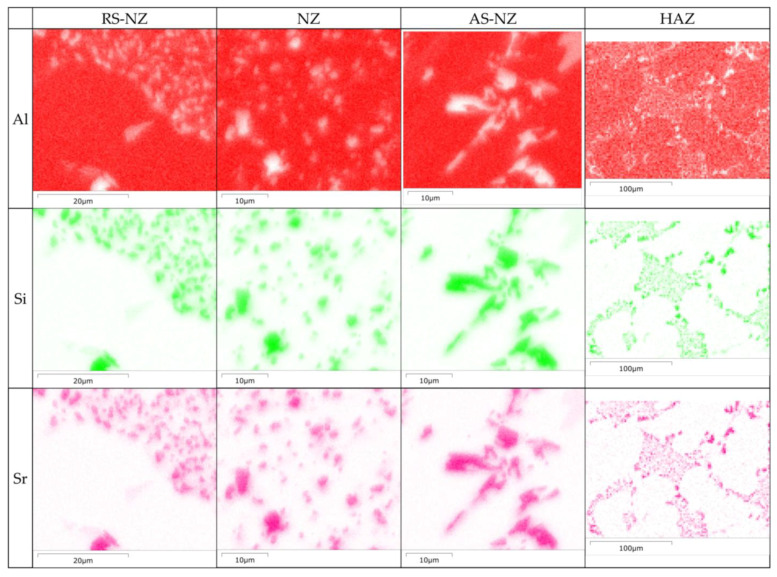

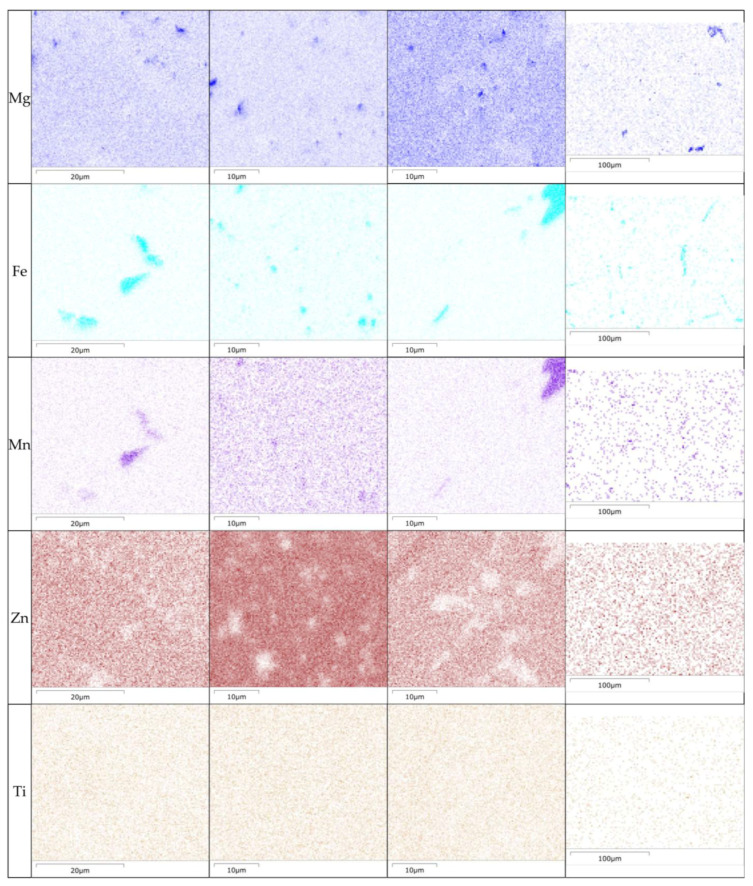
EDS maps of Al, Si, Sr, Mg, Fe, Zn and Ti of different regions of cross-sectional microstructures of the FSWed joint of Al-Si-Mg-Fe-6.5%Zn.

**Figure A4:**
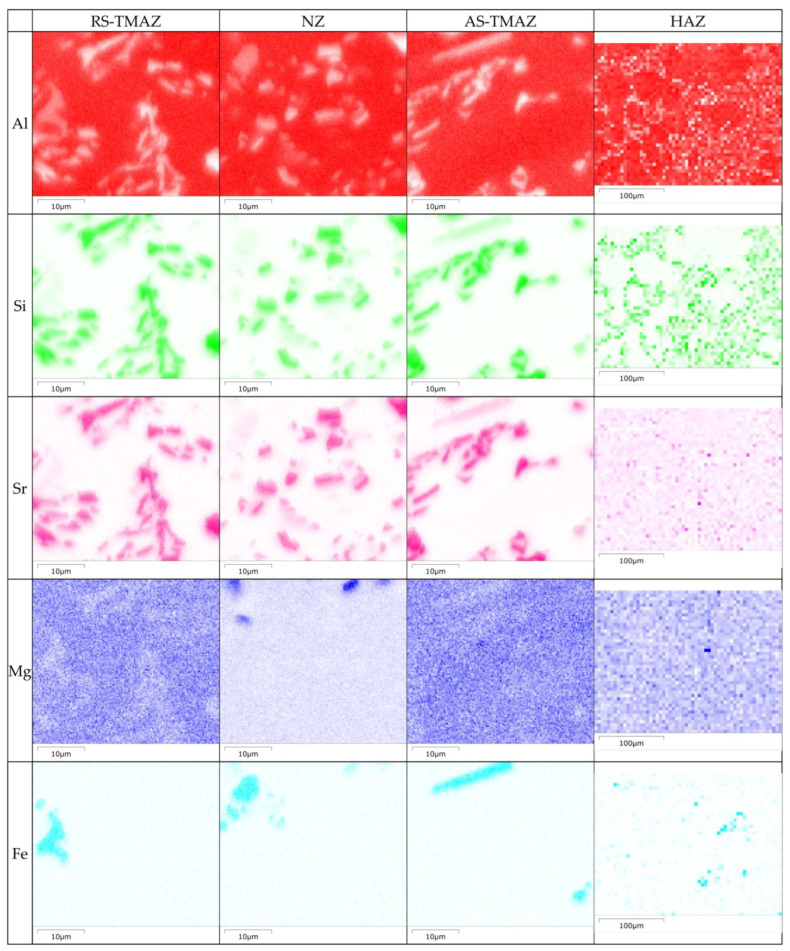

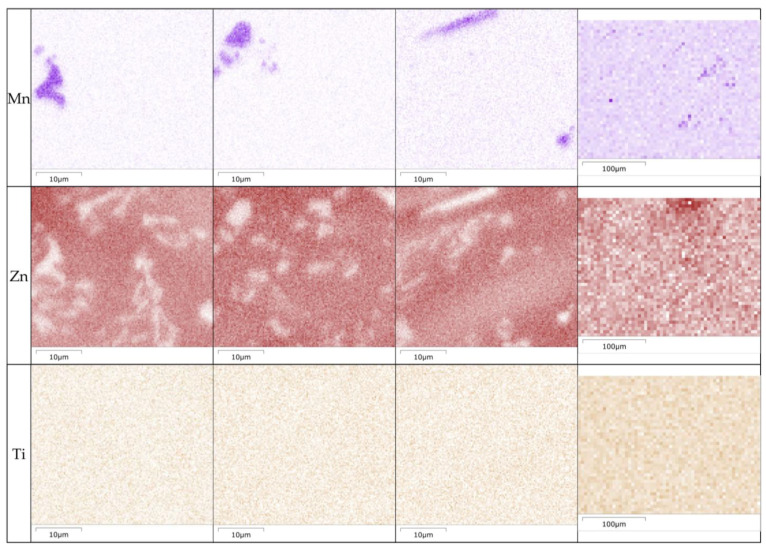
EDS maps of Al, Si, Sr, Mg, Fe, Zn, and Ti of different regions of cross-sectional microstructures of the FSWed joint of Al-Si-Mg-Fe-8.3%Zn.

## Data Availability

The original contributions presented in the study are included in the article, further inquiries can be directed to the corresponding author.

## Abbreviations

The following abbreviations are used in this manuscript:

ASTM	American Society of Testing and Materials
BIW	Boby in white
CE	Counter electrode
CR	Corrosion resistance
DSC	Differential thermal analysis
EBSD	Electron backscattered diffraction
Ecorr	Corrosion potential
EDX	Energy dispersive X-ray spectrometer
EIS	Electrochemical impedance spectroscopy
Epit	Pitting potential
FSW	Friction stir welding
HAZ	Heat-affected zone
HV	Vickers hardness
Icorr	Corrosion current density
KAM	Kernel average misorientation
NZ	nugget zone
OCP	Open circuit potential
RE	Reference electrode
SCE	Saturated calomel electrode
SEM	Scanning electron microscope
TEM	Transmission electron microscopy
TMAZ	Thermo-mechanically affected zone
UTS	Ultimate tensile stress
WE	Working electrode
XPS	X-ray photoelectron spectroscopy
XRD	X-ray diffractometer
YS	Yield stress

## Appendix A

This appendix shows more details of the microstructure of the as-cast alloys and FSWed joints.
